# Increased uterine androgen receptor protein abundance results in implantation and mitochondrial defects in pregnant rats with hyperandrogenism and insulin resistance

**DOI:** 10.1007/s00109-021-02104-z

**Published:** 2021-06-28

**Authors:** Yuehui Zhang, Min Hu, Fan Yang, Yizhuo Zhang, Shuting Ma, Dongqi Zhang, Xu Wang, Amanda Nancy Sferruzzi-Perri, Xiaoke Wu, Mats Brännström, Linus R. Shao, Håkan Billig

**Affiliations:** 1 Department of Obstetrics and Gynecology, Key Laboratory and Unit of Infertility in Chinese Medicine, First Affiliated Hospital, Heilongjiang University of Chinese Medicine, Harbin, 150040 China; 2 Department of Physiology/Endocrinology, Institute of Neuroscience and Physiology, The Sahlgrenska Academy, University of Gothenburg, Medicinaregatan 11, P. O. Box 434, 40530 Gothenburg, Sweden; 3 Department of Traditional Chinese Medicine, The First Affiliated Hospital of Guangzhou Medical University, Guangzhou, 510120 China; 4 Institute of Integrated Traditional Chinese Medicine and Western Medicine, Guangzhou Medical University, Guangzhou, 510120 China; 5 Centre for Trophoblast Research, Department of Physiology, Development and Neuroscience, University of Cambridge, Cambridge, CB2 3EG UK; 6 Department of Obstetrics and Gynecology, Sahlgrenska University Hospital, Sahlgrenska Academy, University of Gothenburg, 41345 Gothenburg, Sweden

**Keywords:** Androgen receptor, Flutamide, Implantation, Mitochondrial function, Pregnant uterus, Polycystic ovary syndrome

## Abstract

**Abstract:**

In this study, we show that during normal rat pregnancy, there is a gestational stage-dependent decrease in androgen receptor (AR) abundance in the gravid uterus and that this is correlated with the differential expression of endometrial receptivity and decidualization genes during early and mid-gestation. In contrast, exposure to 5α-dihydrotestosterone (DHT) and insulin (INS) or DHT alone significantly increased AR protein levels in the uterus in association with the aberrant expression of endometrial receptivity and decidualization genes, as well as disrupted implantation. Next, we assessed the functional relevance of the androgen-AR axis in the uterus for reproductive outcomes by treating normal pregnant rats and pregnant rats exposed to DHT and INS with the anti-androgen flutamide. We found that AR blockage using flutamide largely attenuated the DHT and INS-induced maternal endocrine, metabolic, and fertility impairments in pregnant rats in association with suppressed induction of uterine AR protein abundance and androgen-regulated response protein and normalized expression of several endometrial receptivity and decidualization genes. Further, blockade of AR normalized the expression of the mitochondrial biogenesis marker Nrf1 and the mitochondrial functional proteins Complexes I and II, VDAC, and PHB1. However, flutamide treatment did not rescue the compromised mitochondrial structure resulting from co-exposure to DHT and INS. These results demonstrate that functional AR protein is an important factor for gravid uterine function. Impairments in the uterine androgen-AR axis are accompanied by decreased endometrial receptivity, decidualization, and mitochondrial dysfunction, which might contribute to abnormal implantation in pregnant PCOS patients with compromised pregnancy outcomes and subfertility.

**Key messages:**

- The proper regulation of uterine androgen receptor (AR) contributes to a normal pregnancy process, whereas the aberrant regulation of uterine AR might be linked to polycystic ovary syndrome (PCOS)-induced pregnancy-related complications.
- In the current study, we found that during normal rat pregnancy there is a stage-dependent decrease in AR abundance in the gravid uterus and that this is correlated with the differential expression of the endometrial receptivity and decidualization genes *Spp1*, *Prl*, *Igfbp1*, and *Hbegf*.
- Pregnant rats exposed to 5α-dihydrotestosterone (DHT) and insulin (INS) or to DHT alone show elevated uterine AR protein abundance and implantation failure related to the aberrant expression of genes involved in endometrial receptivity and decidualization in early to mid-gestation.
- Treatment with the anti-androgen flutamide, starting from pre-implantation, effectively prevents DHT + INS-induced defects in endometrial receptivity and decidualization gene expression, restores uterine mitochondrial homeostasis, and increases the pregnancy rate and the numbers of viable fetuses.
- This study adds to our understanding of the mechanisms underlying poor pregnancy outcomes in PCOS patients and the possible therapeutic use of anti-androgens, including flutamide, after spontaneous conception.

**Supplementary Information:**

The online version contains supplementary material available at 10.1007/s00109-021-02104-z.

## Introduction

Polycystic ovary syndrome (PCOS) is one of the most common gynecological disorders associated with fertility difficulties, and it affects 5–25% of all adolescent and reproductive-aged women across multiple geographic ancestries and ethnicities [1–3]. It has a multifactorial etiology and is characterized by reproductive dysfunction accompanied by metabolic abnormalities, and women with PCOS have an increased risk for pregnancy-related complications [4–6]. The etiologies of PCOS are not fully understood, but several possible hypotheses for the uterine manifestations observed in PCOS patients have been postulated to involve hyperandrogenism (androgen excess) together with obesity-related insulin resistance [6–8]. The majority of women with PCOS have attempted or achieved pregnancy. However, data from clinical observational studies indicate that early pregnancy loss, both in spontaneous and in vitro fertilization-induced conceptions, is more common in PCOS patients than in healthy women [9, 10]. Moreover, the incidence of pregnancy loss and infertility in PCOS patients with androgen excess is positively associated with the incidence of pregnancy loss in PCOS patients with insulin resistance [11]. Moreover, Dokras et al. previously reported that hyperinsulinemic states are positively correlated with ovarian androgen levels in women during pregnancy [12]. Therefore, in addition to their separate roles, the coordinated roles of maternal hyperandrogenism and insulin resistance may also account for the early pregnancy loss in PCOS patients. In addition, infertile PCOS patients may experience psychological distresses such as anxiety and depression [13]. These circumstances emphasize the need for a better understanding of the causes of PCOS-induced pregnancy loss and infertility.

The appropriate regulation of the endometrium, a dynamic mucosa layer of the uterus, is essential for embryo implantation during pregnancy [14–16]. The uterine endometrium consists of luminal and glandular epithelial cells, stromal fibroblasts, and vascular and immune cells that are involved in ovarian steroid hormone (17β-estradiol and progesterone)-orchestrated structural and signaling events, including endometrial receptivity, implantation, and decidualization [14, 17]. Moreover, extensive evidence from gene expression studies and transgenic mouse models indicates that aberrant alterations of endometrial epithelia and/or stroma-specific molecules and factors, as well as their interactions, can disturb these pregnancy-related processes, which subsequently might lead to implantation failure and fetal loss [15, 16, 18]. The myometrium is able to synthesize androgens such as testosterone and nonaromatizable 5α-dihydrotestosterone (DHT) during normal pregnancy [19]. However, whether and how the activity of the myometrium, the thickest layer of the uterus, contributes to successful embryo implantation is far from clear.

A recent Swedish nationwide register-based cohort study indicated that early initiation of anti-androgen treatment is correlated with a greater chance of childbirth in PCOS patients after spontaneous conception [20]. However, the underlying mechanisms behind anti-androgen actions in pregnant PCOS patients were unclear. Common opinion holds that the concerted actions of androgens are mostly, but not completely, mediated through binding to nuclear androgen receptor (AR), which belongs to a superfamily of ligand-induced transcription factors [21]. An increasing body of pre-clinical and clinical evidence supports the idea that the androgen-AR axis plays an important role in normal uterine function for both cycling and pregnant females [22, 23]. For example, in humans, circulating androgen levels peak around the time of ovulation, and AR mRNA and protein are predominantly localized to stromal cells in the endometrium and are regulated throughout the menstrual cycle [23, 24]. Findings from clinical observational studies indicate that circulating androgen levels are higher in PCOS patients than non-PCOS women during pregnancy [25, 26], suggesting a further role for androgens in females in the disease state. Of note, treatment with the anti-androgen flutamide decreases clinical hyperandrogenism and improves menstrual cycle regularity and ovulation in affected women [27]. In addition, there are perturbations in endometrial AR expression that parallel the impairment of endometrial function and the subsequent progression of the hallmarks of PCOS [7, 24, 28]. However, whether an aberrant or dysfunctional androgen-AR axis is a cause or consequence of the pathogenesis of PCOS remains speculative.

In rats, we have recently demonstrated that mid-gestational exposure to DHT and insulin (INS) produces a PCOS-like phenotype (i.e., hyperandrogenism and insulin resistance) with increased fetal loss [29, 30]. We show that the dysregulation of implantation and decidualization-related gene expression and ferroptosis is involved in gravid uterine defects in pregnant rats co-exposed to DHT and INS [29, 31]. Given that mitochondrial defects might be an interesting additional mechanism that could potentially contribute to the pathophysiology of PCOS [32], we and others have reported that mitochondrial dysfunction-triggered oxidative stress is significantly increased in PCOS patients who have recurrent miscarriage [33, 34], as well as in PCOS-like rodents [29, 30, 35, 36]. Further, in-utero exposure to flutamide significantly alters the mitochondrial-dependent apoptotic program in rat AR-positive testicular Sertoli and germ cells [37, 38], although no evidence for this is reported in female reproductive tissues. Taken together, these previous studies support the hypothesis that gravid uterine dysfunction can induce PCOS-induced pregnancy-related complications via AR-dependent regulation of implantation and mitochondrial functioning.

In the present study, we evaluated the spatial and temporal cellular expression patterns of the AR protein in relation to endometrial receptivity and decidualization-related gene expression in rats exposed to DHT and/or INS during gestation. To determine the functional relevance of the androgen-AR axis in the gravid uterus, we exposed normal pregnant rats and pregnant rats co-exposed to DHT and INS to flutamide and assessed changes in uterine morphology, endometrial receptivity and decidualization-related gene expression, and mitochondrial morphology and functional markers, as well as maternal metabolism and fertility. Our findings emphasize the complexity of the relationship between AR protein abundance and endometrial receptivity and decidualization-related gene expression and suggest that impairments in the uterine androgen-AR axis are accompanied by decreased endometrial receptivity, decidualization, and mitochondrial function. Moreover, these impairments likely contribute to the subfertility and compromised pregnancy outcomes seen in PCOS patients.

## Materials and methods

### Materials, animal care, and experimental protocols

All experiments complied with the ARRIVE guidelines 2.0 (updated guidelines for reporting animal research) [39]. Adult Sprague–Dawley female (*n* = 72) and male (*n* = 36) rats were obtained from the Laboratory Animal Centre of Harbin Medical University, Harbin, China. On arrival, all animals were maintained in an environmentally controlled and pathogen-free barrier facility on a standard 12 h light/12 h dark cycle at 22 ± 2 °C and 55–65% humidity and with free access to normal diet and water. All rats were used at 70 days of age. Before any experiment was performed, female rats were allowed to acclimatize for a minimum of 7 days and then were monitored daily by vaginal lavage to determine the stage of the estrous cycle [40, 41]. Only rats shown to be in regular estrous cycles were selected for mating experiments. Successful mating was confirmed by microscopic analysis of vaginal smears for the presence of sperm and the appearance of a vaginal plug (designated as gestational day (GD) 0.5). Mated females were randomly assigned to the different treatment groups after excluding for other confounding factors such as body condition and weight as described previously [29, 30].

### Experimental design

#### Experiment 1

**Fig. 1:**
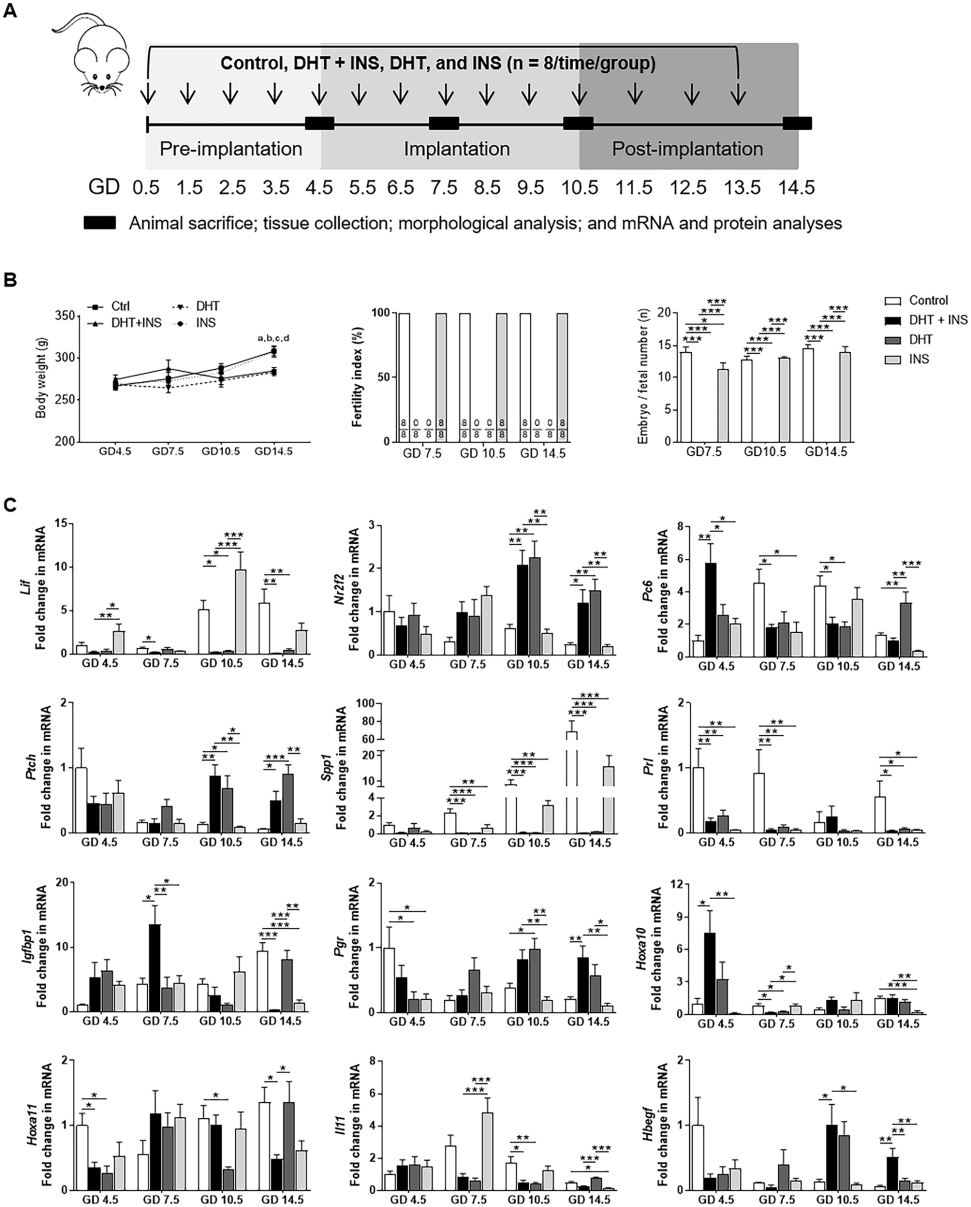
Exposure to DHT or INS alone or in combination alters body weight, embryo or fetal number, and implantation-related gene expression in rats during pregnancy. Schematic of the experimental design **A**. Comparison of body weight and embryo or fetal number in pregnant rats treated with DHT and/or INS (**B**, *n* = 8/group). The fertility index is the percentage of matings that resulted in pregnancy. In all plots the data are presented as means ± SEM. ^a^*P* < 0.05, Control (vehicle) group vs. DHT + INS group; ^b^*P* < 0.01, Control group vs. DHT group; ^c^*P* < 0.05, DHT + INS group vs. INS group; ^d^*P* < 0.05, DHT group vs. INS group. *P < 0.05, ***P < 0.001. Time-dependent changes in implantation-related gene expression **C**. After removing the embryos/fetuses and placenta, uterine tissues from pregnant rats treated with vehicle, DHT + INS, DHT, or INS were used for analyzing genes for uterine receptivity and decidualization by qPCR (*n* = 4–6/group). In all plots, data are presented as means ± SEM (vs. Control GD 4.5 values). Statistical tests are described in the “Materials and methods” section, and differences between the groups are reported as *P < 0.05, ** *P* < 0.01, and ***P < 0.001

For the time-course studies, pregnant rats were randomly assigned to be intraperitoneally injected daily from GD 0.5 with DHT (1.66 mg/kg/day, suspended in sesame oil, Sigma-Aldrich, St. Louis, MO, USA) and/or INS (6.0 IU/day, human recombinant INS diluted in sterile saline, Eli Lilly Pharmaceuticals, Giza, Egypt) or an equal volume of saline and sesame oil as controls. This generated the following four study groups (*n* = 8/group) for comparison: control, DHT + INS, DHT, and INS. The treatment regime is shown in Fig. 1A, and the rationale for the doses of DHT and INS has been previously described [29–31, 36, 41]. Chronic exposure of pregnant rats to DHT and INS results in hyperandrogenism and insulin resistance [29, 30], which is equivalent to that found in PCOS patients [25, 26, 42, 43]. All animals were exposed to isoflurane (2% in a 1:1 mixture of oxygen or air, RWD Life Science Co., Shenzhen, China) followed by exsanguination. Rats were sacrificed on GD 4.5, 7.5, 10.5, and 14.5. The reason for choosing GD 4.5 to GD 14.5 was the necessity to follow the physiological and cellular events such as endometrial receptivity, decidualization, and embryo implantation that occur in rodents during early and mid-gestation [18]. If implanted embryos were found, a stereomicroscope was used to separate fetal and placental tissues from the uterine wall. Uterine tissues were dissected, weighed, and immediately frozen in liquid nitrogen and stored at − 70 °C for quantitative real-time PCR (qPCR) and Western blot analyses or fixed for morphological and immunohistochemical analyses.

#### Experiment 2

For pharmacological studies, the anti-androgen flutamide (25 mg/kg/day, suspended in 100 µl sesame oil, F-9397, Sigma-Aldrich) [44, 45] or vehicle (100 µl sesame oil) was intraperitoneally injected daily from GD 0.5 in control and DHT + INS-exposed pregnant rats (*n* = 20/group). The flutamide dose was specifically chosen as it has been found to effectively improve DHT-induced defects in ovarian morphology and to restore reproductive cycles in a PCOS-like rodent model [46]. The oral glucose tolerance test (OGTT) was assessed in pregnant rats on GD 13.5, and animals were allowed to recover overnight before blood collection. On GD 14.5, all rats were exposed to isoflurane (2% in a 1:1 mixture of oxygen or air, RWD Life Science Co.), followed by exsanguination. Trunk blood was collected directly from the heart and maintained at room temperature for 1 h before isolation of serum. Following sacrifice, the uterus was dissected, the number of implanted embryos was recorded, and the fetal and placental tissues were separated from the uterine wall under a stereomicroscope. Further, uterine tissues were dissected, weighed, and immediately frozen in liquid nitrogen and stored at −70 °C for qPCR and Western blot analyses or fixed for morphological and immunochemical analyses.

### OGTT

Glucose tolerance was assessed in pregnant rats on GD 13.5 using an OGTT as described previously [29, 30]. Briefly, rats were fasted for 10 h and blood glucose concentrations were determined at 0, 30, 60, 90, and 120 min after D-glucose administration (3 g/kg body weight in saline, oral). Glucose concentrations were measured using a hand-held glucometer from blood sampled from the tail vein.

### RNA isolation and qPCR

The isolation and quantification of the RNA and the qPCR assays were performed as previously described [31, 47]. The PCR amplifications were performed with SYBR green qPCR master mix (#K0252, Thermo Scientific, Rockford, IL). Total RNA was prepared from the frozen whole uterine tissues, and single-stranded cDNA was synthesized from each sample (2 μg) with M-MLV reverse transcriptase (#0,000,113,467, Promega Corporation, Fitchburg, WI) and RNase inhibitor (40 U) (#00,314,959, Thermo Scientific). cDNA (1 μl) was added to a reaction master mix (10 μl) containing 2 × SYBR green qPCR reaction mix (Thermo Scientific) and gene-specific primers (5 μM of forward and reverse primers). All reactions were performed at least twice, and each reaction included a nontemplate control. Specific sample sizes are denoted in the figure legends. Several housekeeping genes, including *Gapdh*, *Actb* (*β-actin*), and *L19*, were tested before analysis. However, only *Gapdh* and *Actb* were stably expressed between the groups and thus used as the reference gene for our analysis. Fold changes in mRNA expression were calculated by the ΔΔCT method using *Gapdh* and *Actb* as the endogenous controls, and the results are expressed as fold changes after normalizing to the controls. The qPCR primers used in this study are listed in Supplemental Table 1. All sets of primers were validated for qPCR prior to analysis. This involved determining that the efficiency of amplification using a standard curve of cDNA was above 85% and not different from the *Gapdh* and *Actb* reference genes, and there were no nonspecific PCR products seen in a melting curve analysis immediately after the amplification or in parallel reactions with un-transcribed RNA or in reactions without templates (the negative controls).

**Table 1:** Pearson correlation coefficients for the relation of AR protein abundance, implantation-related genes, and uterine cell marker protein expression in the gravid uterus of control pregnant rats

AR	Coefficient, *r*	*p* value
Implantation-related genes		
*Lif*	−0.39	0.08
*Nr2f2*	0.31	0.17
*Pc6*	0.06	0.81
*Ptch*	0.41	**0.05**
*Spp1*	−0.46	**0.04**
*Prl*	0.56	**0.01**
*Igfbp1*	−0.54	**0.02**
*Pgr*	0.38	0.08
*Hoxa10*	−0.16	0.49
*Hoxa11*	−0.25	0.28
*Il11*	0.10	0.66
*Hbegf*	0.46	**0.04**
Uterine cell marker proteins		
pan-Cytokeratin	0.03	0.88
Vimentin	0.69	** < 0.01**
α-SMA	0.59	** < 0.01**

*n* = 5–6/GD

*AR* androgen receptor, *Lif* leukemia inhibitory factor, *Nr2f2* nuclear receptor subfamily 2 group F member 2, *Pc6* protein convertase 5/6, *Ptch* patched, *Spp1* osteopontin/secreted phosphoprotein 1, *Prl* prolactin, *Igfbp1* insulin-like growth factor binding protein 1, *Pgr* progesterone receptor, *Hoxa10* homeobox A10, *Il11* interleukin-11, *Hbegf* heparin-binding EGF-like growth factor, *α-SMA* α-smooth muscle actin

### Protein isolation and Western blot analysis

The extraction of total protein and Western blot analysis was conducted with standard protocols as previously described [31, 48]. Uterine tissue proteins were isolated by homogenization in radioimmunoprecipitation assay buffer (Sigma-Aldrich) supplemented with cOmplete Mini protease inhibitor cocktail tablets (Roche Diagnostics, Mannheim, Germany) and PhosSTOP phosphatase inhibitor cocktail tablets (Roche Diagnostics). After determining the total protein concentration by Bradford protein assay (Thermo Fisher Scientific), equal amounts (30 μg) of protein were resolved on 4–20% TGX stain-free gels (Bio-Rad Laboratories GmbH, Munich, Germany) and transferred onto polyvinylidene fluoride membranes. The membranes were probed with different primary antibodies (Supplemental Table 2) in 0.01 M Tris-buffered saline supplemented with Tween 20 containing 5% w/v nonfat dry milk followed by anti-rabbit IgG horseradish peroxidase (HRP)-conjugated goat (A0545) or anti-mouse IgG HRP-conjugated goat (A2304) secondary antibody (1:1000 dilution, Sigma-Aldrich). Signal was detected using the SuperSignal West Dura Extended Duration Substrate (Thermo Fisher Scientific) and captured using a ChemiDoc MP Imaging System (Bio-Rad). For each Western blot, ultraviolet activation of the Criterion stain-free gel was used to assess total protein loading for each sample. Band densitometry was performed using Image Laboratory (Version 5.0, Bio-Rad), and the intensity of each protein band was normalized to the total protein in the individual sample for calculating protein abundance data. Due to the number of samples per group, multiple gels were run per group. For quantification and to ensure standardization across blots, the abundance of the target protein was normalized to the mean value for the control group on the blot, and then all of the normalized values were compared in order to assess the effects of the treatments. This ensured the accurate comparison of protein abundance across groups with the single tissue sample.

### Histological examination and immunohistochemical staining

Histological processing and immunohistochemistry were performed according to previously described methods [47, 48]. The 4% formaldehyde-fixed uterine tissues were subjected to paraffin embedding and sectioned at 5 μm. Tissue sections were stained with hematoxylin and eosin (H&E) or with immunohistochemistry. After heat-induced antigen retrieval by pressure cooking in citrate buffer (pH 6), endogenous peroxidase activity was quenched by incubation with 3% hydrogen peroxide in phosphate-buffered saline (PBS) for 10 min, and then nonspecific binding was blocked with 10% normal goat serum for 1 h at room temperature. Tissue sections were incubated with the primary antibody against AR (Supplemental Table 2) overnight at 4 °C in a humidified chamber. Afterward, slides were stained using the avidin-biotinylated-peroxidase ABC kit followed by a 5-min treatment with 3,3′-diaminobenzidine (DAB, SK-4100, Vector Laboratories). The optimal concentration of anti-AR antibody (1:200) was determined in initial experiments, and background settings were adjusted from the examination of negative control specimens prepared using a rabbit IgG antibody (Supplemental Fig. 1.1–1.6). The anti-AR antibody was also initially characterized for specificity using human prostatic tissues and cell lines and in rat testis tissues by Western blotting and immunofluorescence analyses according to the manufacturer’s instructions (https://www.abcam.com/androgen-receptor-antibody-epr15352-ab133273.html), and no specific immunoreactivity was detected in neighboring tissue sections (Supplemental Figs. 2.6 and 4.7–4.8). Stained samples were observed and imaged on a Nikon E-1000 microscope (Japan) under bright-field optics and photomicrographed using Easy Image 1 (Bergström Instrument AB, Sweden). All images were taken with precisely the same parameters for all experimental groups.

### TEM

Transmission electron microscopy (TEM) was performed according to published reports [30, 31]. Briefly, fresh uterine tissues were fixed in 2.5% glutaraldehyde in phosphate-buffered saline (PBS, pH 7.2–7.4) for 1 h at room temperature and further rinsed with 0.1 M PBS three times for 15 min each. The tissues then underwent permeation and dehydration, and samples were finally embedded in Epon epoxy resin. The 50–60 nm sections were post-stained with 3% uranyl acetate and lead citrate and were viewed using a transmission electron microscope (H-7650, Hitachi, Japan) equipped with an electron imaging spectrometer. Image collection and parameter settings were identical for each of the different tissues/regions analyzed.

### Measurement of biochemical parameters

The concentrations of serum hormones (total testosterone (T), androstenedione (A4), DHT, dehydroepiandrosterone (DHEA), sex hormone-binding globulin (SHBG), progesterone (P4), 17β-estradiol (E2), and fasting INS) were quantified using chemiluminescence (Beckman Coulter, Inc., CA, and Abbott Laboratories, IL). The reproducibility (intra/inter-assay coefficients of variation) of rat T, A4, DHT, DHEA, SHBG, P4, E2, and INS were 6.2%/6.6%, 6.7%/6.9%, 6.2%/6.7%, 6.3%/6.6%, 6.4%/6.8%, 6.4%/6.6%, 6.5%/6.8%, and 6.3%/6.6%, respectively.

### Statistical analysis

No statistical methods were used to pre-determine the sample size. Statistical analyses were performed using SPSS version 24.0 for Windows (SPSS Inc., Chicago, IL). The normal distribution of the data was tested with the Shapiro–Wilk test. For the time-course studies, normally distributed data were analyzed by two-way ANOVA (including the factors of GD and hormonal treatment) followed by pairwise Tukey post hoc tests. For the pharmacological studies using the AR antagonist flutamide, normally distributed data were analyzed by one-way ANOVA followed by Tukey post hoc tests. Data that were not normally distributed were tested for statistical significance between groups with the Kruskal–Wallis test. Data are presented as means ± standard error of the mean (SEM), and the sample size (*n*) is listed in the figure legends and indicates the number of pregnant rats in each experiment. All *P*-values less than 0.05 were considered statistically significant. The main effects of GD and/or hormonal treatment are referred to as *P*_GD_, *P*_treatment_, and *P*_GD:treatment_, respectively. Pearson’s correlation coefficient was used to examine the strength of the associations between AR protein expression and the mRNA levels of genes contributing to endometrial receptivity and decidualization in the gravid uterus from control pregnant rats from GD 4.5 to GD 14.5.

## Results

### Regulation of endometrial receptivity and decidualization-related gene expression in pregnant rats in response to different hormonal treatments

As shown in Fig. 1B, age-matched DHT + INS-exposed and DHT-exposed pregnant rats were lighter than controls and INS-exposed pregnant rats at GD 14.5. We found that from GD 7.5 to GD 14.5, the fertility index was the same between controls and INS-exposed pregnant rats. However, DHT + INS-exposed and DHT-exposed pregnant rats had no apparent pregnancies compared to control and INS-exposed pregnant rats. Although maternal exposure to INS significantly reduced the embryo number at GD 7.5 compared to controls, there were no embryos or fetuses found in DHT + INS or DHT-exposed pregnant rats from GD 7.5 to GD 14.5. We therefore determined whether early and mid-gestational exposure to DHT and/or INS affects endometrial receptivity and decidualization-related gene expression in the gravid uterus (Fig. 1C). There were significant interactions between different GDs and treatments in determining the overall gene expression of *Lif*, *Nr2f2*, *Ptch*, *Spp1*, *Prl*, *Hoxa10*, and *Il11* (*P*_GD_ < 0.05, *P*_treatment_ < 0.05, *P*_GD:treatment_ < 0.05). Additionally, GD exerted a significant effect on *Pc6*, *Igfbp1*, *Hoxa11*, and *Hbegf* mRNA expression (*P*_GD_ < 0.05, *P*_GD:treatment_ < 0.05), whereas the hormonal treatment had a significant effect on *Pgr* mRNA expression (*P*_treatment_ < 0.001, *P*_GD:treatment_ < 0.001).

At GD 4.5, all hormonal treatments reduced *Prl* mRNA expression compared to controls but did not affect *Nr2f2*, *Ptch*, *Spp1*, *Igfbp1*, *Il11*, or *Hbegf* mRNA expression (Fig. 1C). Although the *Hoxa11* mRNA level was lower in DHT + INS-exposed and DHT-exposed pregnant rats compared to controls at GD 4.5, we found that *Pc6* and *Hoxa10* mRNAs were increased in DHT + INS-exposed pregnant rats compared to controls and to DHT-exposed and INS-exposed pregnant rats. At GD 7.5, five of the measured mRNAs (*Nr2f2*, *Ptch*, *Pgr*, *Hoxa11*, and *Hbegf*) showed no changes in any of the treatment groups, whereas *Spp1* and *Prl* mRNAs were decreased in pregnant rats exposed to DHT and/or INS compared to controls (Fig. 1C). At the same time, *Lif*, *Pc6*, and *Hoxa10* mRNAs were decreased and *Igfbp1* mRNA expression was increased in DHT + INS-exposed pregnant rats compared to control pregnant rats. At both GD 10.5 and GD 14.5, *Lif* and *Spp1* mRNA expression remained reduced, but *Nr2f2* and *Ptch* mRNAs were increased in DHT + INS-exposed and DHT-exposed pregnant rats compared to controls (Fig. 1C). We did not find any significant differences in *Prl*, *Igfbp1*, or *Hoxa10* mRNA expression in response to the hormonal treatments at GD 10.5, but these three genes were decreased in pregnant rats exposed to DHT and/or INS compared to control pregnant rats at GD 14.5. In addition, maternal exposure to DHT alone or together with INS decreased *Pc6*, *Hoxa11*, and *Il11* mRNA expression at GD 10.5, while *Pgr* and *Hbegf* mRNAs were increased at this GD. At GD 14.5, we also found increased *Pgr* and *Hbegf* mRNAs and decreased *Hoxa11* mRNA in DHT + INS-exposed pregnant rats compared to control pregnant rats. These findings suggest that there are stage-dependent alterations in endometrial receptivity and decidualization-related gene expression in the gravid uterus during normal pregnancy. Moreover, these can be influenced by maternal exposure to DHT and INS and are related to implantation failure during gestation.

### Distinctive patterns of uterine AR expression and localization in pregnant rats in response to different hormonal treatments

**Fig. 2:**
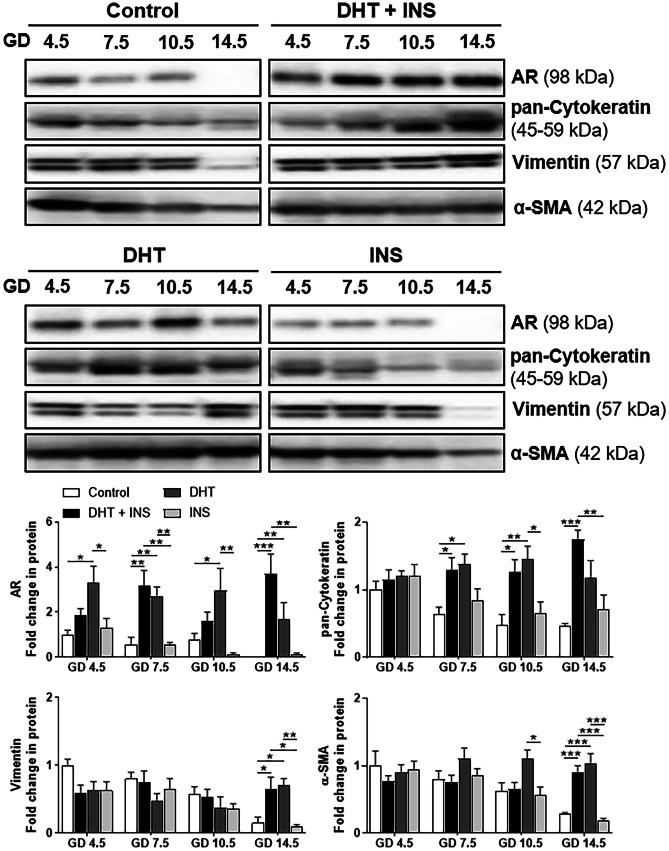
Differential regulation of AR protein in uteri collected from pregnant rats exposed to DHT and/or INS from GD 4.5 to GD 14.5. Time-dependent regulation of AR protein abundance in the pregnant uterus. After removing the embryos/fetuses and placenta, the uterine tissues from pregnant rats treated with control (vehicle), DHT + INS, DHT, or INS were used for analyzing AR and uterine cell marker proteins (cytokeratin, vimentin, and α-smooth muscle actin) by Western blotting. In all plots, the relative mean protein abundance ± SEM (vs. Control GD 4.5 values, *n* = 5–6/group) was measured by Western blotting with total proteins serving as loading controls. Statistical tests are described in the “Materials and methods” section, and differences between the groups are reported as *P < 0.05, **P < 0.01, and ***P < 0.001. The size representation in kilodaltons (kDa), as determined by a molecular weight ladder, is shown to the right

We then sought to determine whether gestational-age dependent endometrial receptivity and decidualization-related gene expression might be related to AR expression in the gravid uterus and if aberrant uterine AR expression is occurring in pregnant rats exposed to DHT and/or INS. Using Western blotting (Fig. 2), we found that exposure to DHT + INS and to DHT alone significantly affected uterine AR abundance from GD 4.5 to GD 14.5 (*P*_treatment_ < 0.001, *P*_GD:treatment_ = 0.023). In particular, uterine AR abundance was increased in DHT-exposed pregnant rats compared to controls at all GDs. However, a similar increase in AR abundance was only observed in DHT + INS-exposed pregnant rats compared to controls at GD 7.5 and GD 14.5. There was no significant difference in AR abundance between control and INS-exposed pregnant rats at any GD (Fig. 2).

**Fig. 3:**
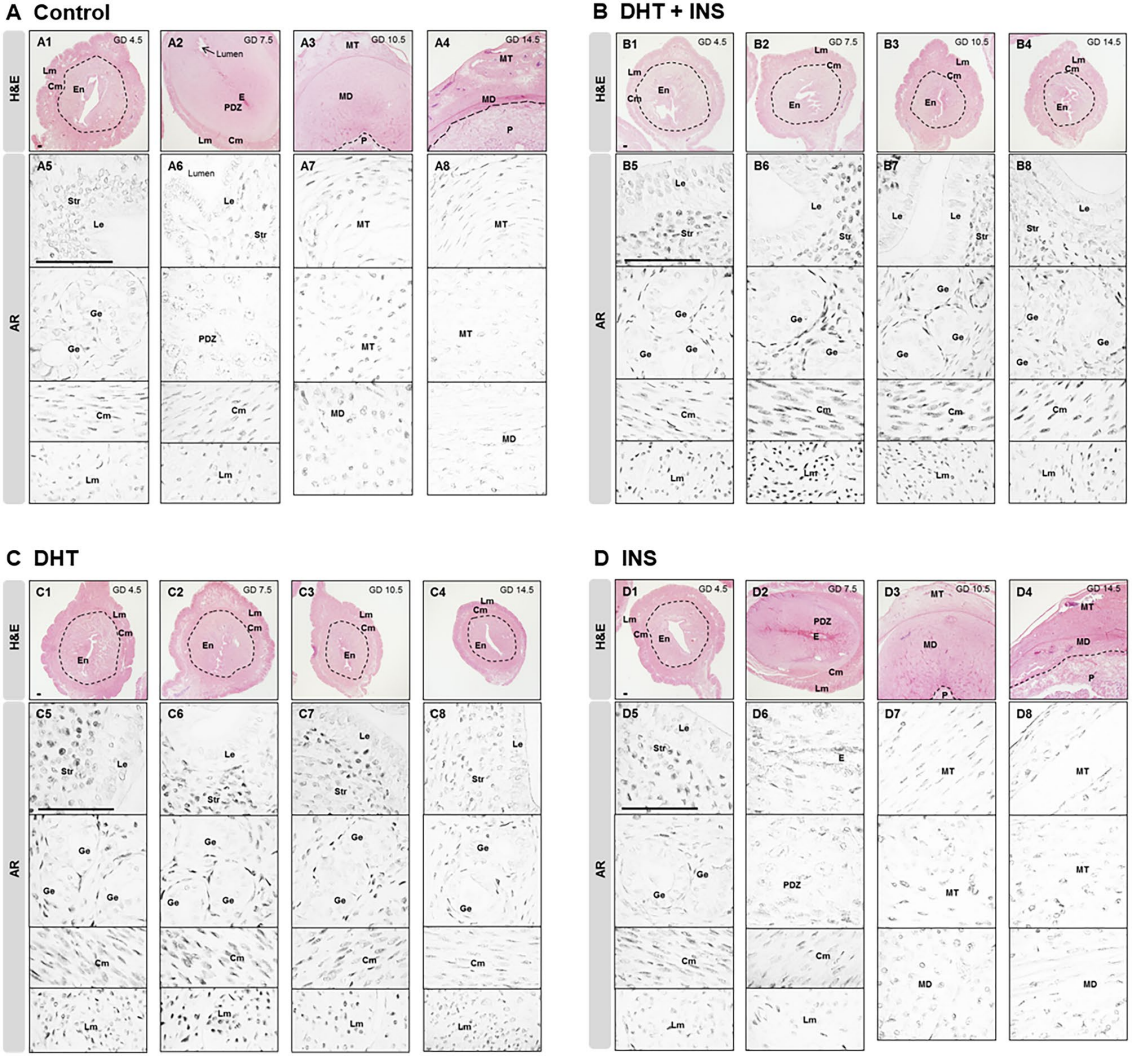
Localization of the AR protein in uteri collected from pregnant rats exposed to DHT and/or INS from GD 4.5 to GD 14.5. Histological appearance of gravid uteri using hematoxylin and eosin (H&E) staining (A1–A4, B1–B4, C1–C4, and D1–D4) and AR localization by immunohistochemistry (A5–A8, B5–B8, C5–C8, and D5–D8) in pregnant rats treated with vehicle **A**, DHT + INS **B**, DHT **C**, or INS **D**. Images are representative of eight tissue replicates. GD, gestational day; DHT, 5α-dihydrotestosterone; INS, insulin; En, endometrium; Cm, circular myometrium; Lm, longitudinal myometrium; Le, luminal epithelial cells; Ge, glandular epithelial cells; Str, stromal cells; E, embryo; PDZ, primary decidual zone; MT, mesometrial triangle; MD, mesometrial decidua; P, placental disc. Scale bars (100 μm) are indicated in the photomicrographs

Pearson correlation analysis showed that in control pregnant rats, uterine AR abundance was significantly correlated with *Spp1*, *Prl*, *Igfbp1*, and *Hbegf* mRNA expression and with vimentin and α-SMA protein abundance from GD 4.5 to GD 14.5 (Table 1). Western blotting showed that the abundance of cytokeratin, which is highly abundant in endometrial epithelial cells, was increased in DHT + INS-exposed and DHT-exposed pregnant rats compared to controls from GD 7.5 to GD 14.5 (*P*_treatment_ < 0.001, *P*_GD:treatment_ = 0.016), while the abundance of vimentin, which is mainly in endometrial stromal cells (*P*_GD_ = 0.001, *P*_GD:treatment_ = 0.003), and α-smooth muscle actin, which is mainly in uterine myometrial cells (*P*_GD_ = 0.002, *P*_treatment_ < 0.001, *P*_GD:treatment_ = 0.002), were increased in DHT + INS-exposed and DHT-exposed pregnant rats compared to controls at GD 14.5 (Fig. 2).

According to the GD-dependent structural changes in the rat uterus [49, 50], we found that from GD 4.5 to GD 14.5, the development of the gravid uterus was histo-morphologically normal in controls (Fig. 3A1–A4, and Suppl Figs. 2–4) and was similar in INS-exposed pregnant rats (Fig. 3D1–D4). In contrast, we found that compared to control and INS-exposed pregnant rats, DHT + INS-exposed and DHT-exposed pregnant rats contained endometrial and myometrial compartments at all stages. With increasing GD, the gravid uterine diameters (middle zone — endometrial compartment) were decreased by exposure to DHT + INS (Fig. 3B1–B4) and to DHT alone (Fig. 3C1–C4). Consistent with studies on human samples [24, 51, 52], immunohistochemical studies in control pregnant rats showed that AR nuclear immunoreactivity was detected in decidual stromal cells and myometrial smooth muscle cells, with lower expression in luminal and gradual epithelial cells at GD 4.5 (Fig. 3A5), GD 7.5 (Fig. 3A6 and Suppl Fig. 2), and GD 10.5 (Fig. 3A7, and Suppl Fig. 3). In addition, the reduction in AR immunoreactivity in the gravid uterus during gestation was found in the mesometrial decidua and triangle compartments in control pregnant rats (Fig. 3A8). A similar loss of cellular AR immunoreactivity during gestation was also seen in the gravid uterus of INS-exposed pregnant rats (except that decidual stromal cells showed higher immunoreactivity at GD 4.5) (Fig. 3D5–D8). Regardless of the GD studied, strong AR immunoreactivity was observed in stromal cells in the DHT + INS-exposed and DHT-exposed gravid uterus (Fig. 3B5–B8 and C5–C8, upper and middle rows). In addition, immunostaining for AR was absent, or very sparse, in luminal and glandular epithelial cells at all GDs in the DHT + INS-exposed and DHT-exposed rats (Fig. 3B5–B8 and C5–C8, upper and middle rows). Despite the stepwise variation of AR immunoreactivities when plotted against GD, AR was distributed homogeneously in smooth muscle cells across the myometrium in DHT + INS-exposed and DHT-exposed pregnant rats (Fig. 3B5–B8 and C5–C8, bottom rows). These findings indicate that the stage-dependent and androgen-specific regulation of uterine AR protein abundance is associated with changes in endometrial functional gene expression.

### Flutamide reversed metabolic and endocrine abnormalities and partially restored fertility in DHT + INS-exposed pregnant rats

**Fig. 4:**
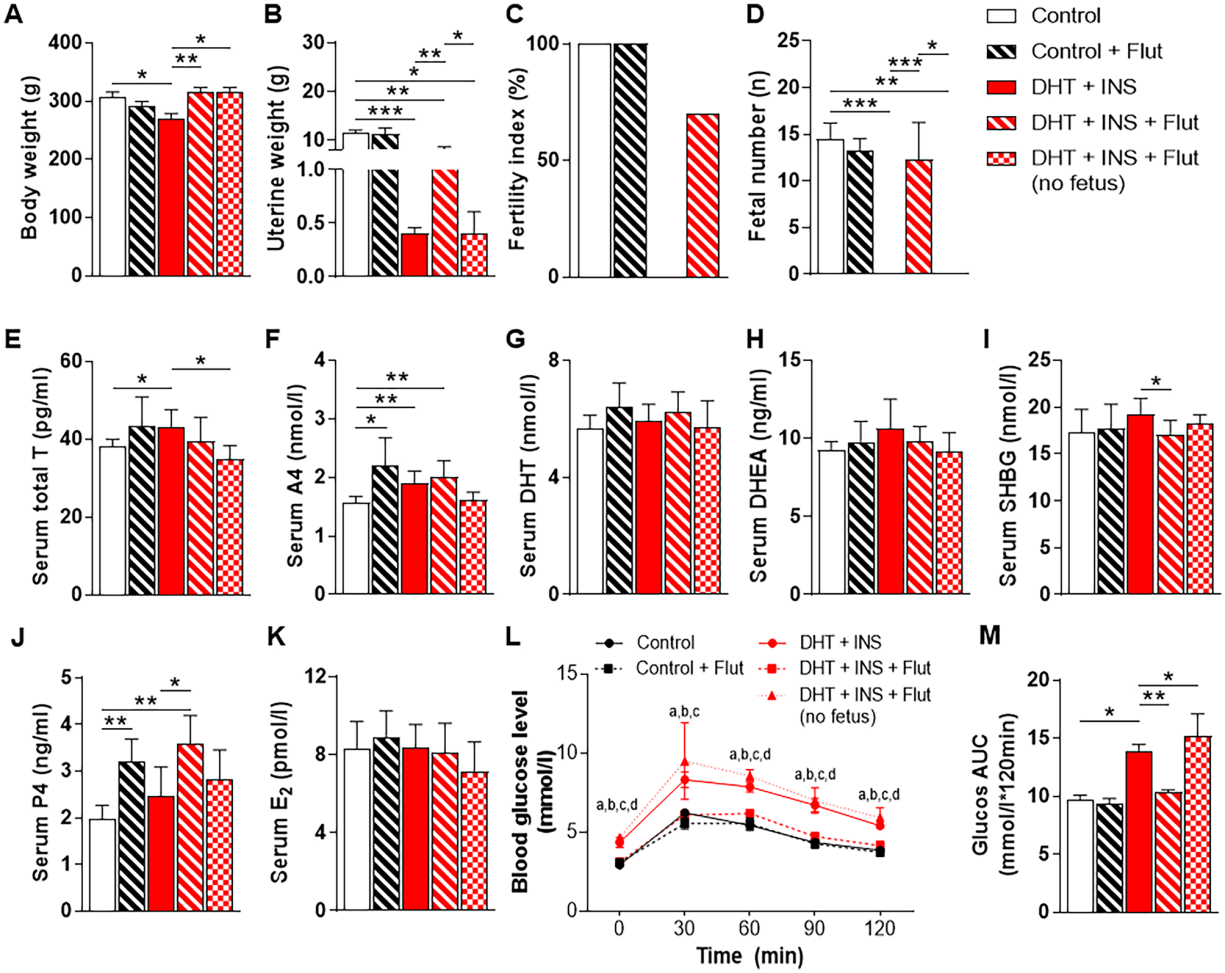
Chronic treatment with flutamide induces changes in hormones and metabolites in control and DHT + INS-exposed pregnant rats at GD 14.5. Comparison of body weight **A**, uterine weight **B**, fertility index **C**, fetal number **D**, serum total T **E**, A4 **F**, DHT **G**, DHEA **H**, SHBG **I**, P4 **J**, E2 **K**, blood glucose concentrations during OGTT **L**, and area under the curve (AUC) for glucose **M** in pregnant rats treated with and without DHT and INS [*n* = 10/group, except for the DHT + INS + flutamide group (with fetuses, *n* = 7) and the DHT + INS + flutamide (without fetuses) group (*n* = 3)]. The fertility index is the percentage of matings that resulted in pregnancy. AUC was calculated by the formula [0.5 × (BG0 + BG30)/2 + 0.5 × (BG30 + BG60)/2 + 0.5 × (BG60 + BG90)/2 + 0.5 × (BG90 + BG120)/2], where the BG terms are the blood glucose levels at 0 min, 30 min, 60 min, 90 min, and 120 min. ^a^*P* < 0.05, control (vehicle) group vs. DHT + INS group; ^b^*P* < 0.05, control group vs. DHT + INS + flutamide (no fetuses) group; ^c^*P* < 0.05, DHT + INS group vs. DHT + INS + flutamide (with fetuses) group; ^d^*P* < 0.05, DHT + INS + flutamide group vs. DHT + INS + flutamide (without fetuses) group. In all plots, data are presented as means ± SEM. Statistical tests are described in the “Materials and methods” section, and differences between the groups are reported as *P < 0.05, **P < 0.01, and ***P < 0.001

Our next goal was to evaluate whether the inhibition of androgen-AR activation with flutamide could rescue the gravid uterine defects and infertility induced by combined exposure to DHT and INS (which mimic the typical PCOS features). Flutamide treatment did not affect body weight, uterine weight, fertility index, or fetal number in control pregnant rats at GD 14.5 (Fig. 4A–D). However, flutamide treatment increased the body weight of DHT + INS-exposed pregnant rats whether they did or did not have fetuses (Fig. 4A). Flutamide also increased uterine weight in DHT + INS-exposed pregnant rats (with fetuses), although this value was still significantly lower than control pregnant rats (Fig. 4B). The improved uterine weight in flutamide-treated DHT + INS-exposed pregnant rats (with fetuses) reflected a 70% increase in pregnancy rate and a similar fetal number compared to control pregnant rats (Fig. 4C, D). In line with the hyperandrogenic conditions, circulating total T and A4 concentrations were increased in DHT + INS-exposed pregnant rats compared to controls (Fig. 4E, F). Treatment with flutamide increased P4 concentration in both control and DHT + INS-exposed pregnant rats (with fetuses) but did not affect P4 concentration in DHT + INS-exposed pregnant rats (without fetuses) (Fig. 4J). There was no effect of flutamide on DHT, DHEA, SHBG, or E2 concentrations in control pregnant rats, but SHBG was reduced by flutamide in DHT + INS-exposed pregnant rats (with fetuses) (Fig. 4G-K). Furthermore, control and flutamide-treated DHT + INS-exposed pregnant rats (with fetuses) were significantly more glucose tolerant compared to DHT + INS-exposed pregnant rats without flutamide treatment (Fig. 4L, M). Of note, even with flutamide treatment, DHT + INS-exposed pregnant rats with fetuses exhibited a greater increase in glucose tolerance compared to DHT + INS-exposed pregnant rats without fetuses (Fig. 4L, M). These data suggest that flutamide treatment partially improves fertility and metabolic aberrations in pregnant rats with hyperandrogenism and insulin resistance.

### Impact of flutamide on uterine morphology, endometrial receptivity and decidualization-related gene expression, and AR and androgen-regulated protein expression

**Fig. 5:**
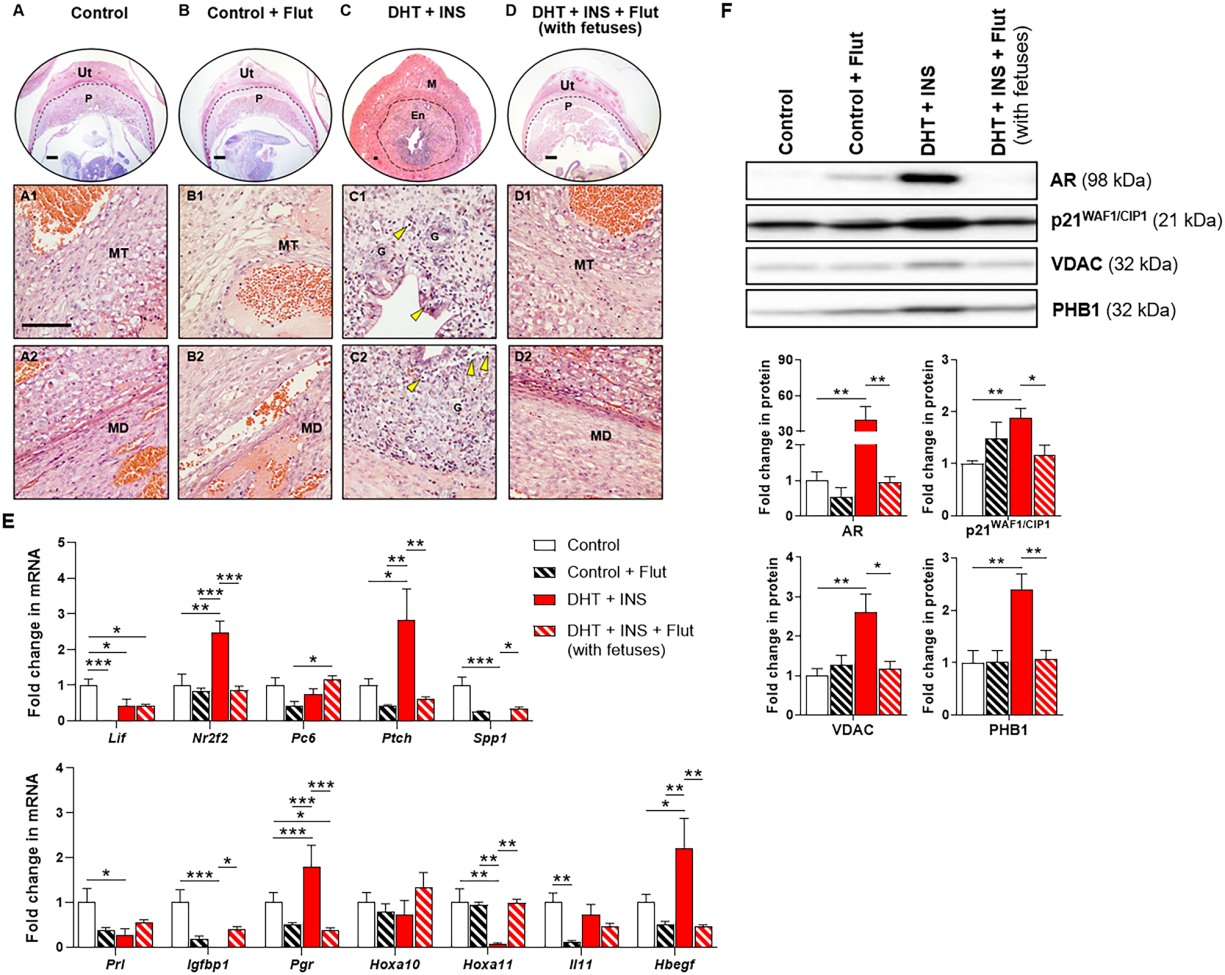
Effects of flutamide on uterine morphology, implantation-related gene expression, and AR, p21^WAF1/CIP1^, and mitochondrial marker protein expression in control and DHT + INS-exposed pregnant rats at GD 14.5. Histological analysis by H&E staining in vehicle control **A**, **B** and DHT + INS-exposed **C**, **D** pregnant rats treated with flutamide **B**, **D**. The yellow arrowheads indicate infiltrated immune cells in the endometrial gland. Images are representative of 7–10 tissue replicates. *Ut*, uterus; *P*, placenta; *MT*, mesometrial triangle; *MD*, mesometrial decidua; *M*, myometrium; *En*, endometrium; *G*, gland. Scale bars (100 μm) are indicated in the photomicrographs. After removing the embryos/fetuses and placentas, uterine tissues from control and DHT + INS-treated pregnant rats treated with flutamide were used for analyzing uterine receptivity and decidualization genes by qPCR (**F**, *n* = 5–7/group), and the relative abundance of AR, p21^WAF1/CIP1^, VDAC, and PHB1 proteins was determined by Western blotting (**G**, 5–6/group). The size representation in kDa, as determined by a molecular weight ladder, is shown to the right. In all plots, data are presented as means ± SEM (vs. control vehicle values). Statistical tests are described in the “Materials and methods” section, and differences between the groups are reported as *P < 0.05, **P < 0.01, and ***P < 0.001

We observed that control and DHT + INS-exposed pregnant rats (with fetuses) treated with flutamide showed similar uterine histo-morphology as untreated control rats (Fig. 5A, B-D). However, in line with our previous observation (Fig. 3B4), the uteruses from DHT + INS-exposed pregnant rats without flutamide treatment showed both endometrial and myometrial compartments (Fig. 5C). Additionally, we found that the numbers and infiltration of immune cells into the luminal and glandular epithelia and the stroma were increased in DHT + INS-exposed pregnant rats without flutamide treatment (Fig. 5C). These histological features were also observed in flutamide-treated DHT + INS-exposed pregnant rats that had no fetuses (Supplemental Fig. 5).

In agreement with previous results (Fig. 1C), there was significant dysregulation of several endometrial receptivity and decidualization-related genes (i.e., *Lif*, *Nr2f2*, *Ptch*, *Spp1*, *Prl*, *Igfbp1*, *Pgr*, *Hoxa11*, and *Hbegf*) between control and DHT + INS-exposed pregnant rats (Fig. 5E). Among these genes, treatment with flutamide decreased *Lif* and *Il11* mRNAs in control pregnant rats (Fig. 5E). However, we found that flutamide treatment decreased *Nr2f2*, *Ptch*, *Pgr*, and *Hbegf* mRNAs and increased *Spp1*, *Igfbp1*, and *Hoxa11* mRNAs in DHT + INS-exposed pregnant rats (with fetuses) (Fig. 5E). Because p21^WAF1/CIP1^ is an androgen-regulated response protein [53], and because the mitochondrial marker proteins (VDAC and PHB1) are dysregulated in the placentas of pregnant rats co-exposed to DHT and INS [30], we sought to determine whether flutamide could regulate the expression of these proteins in the pregnant rat uterus. We observed significantly increased protein abundance of AR, p21^WAF1/CIP1^, VDAC, and PHB1 in DHT + INS-exposed pregnant rats compared to control rats (Fig. 5F). Although no differences in AR, p21^WAF1/CIP1^, VDAC, or PHB1 protein abundance were seen with flutamide treatment in control rats, we found that flutamide significantly decreased the abundance of these proteins in DHT + INS-exposed pregnant rats (with fetuses) compared to DHT + INS-exposed pregnant rats without flutamide treatment (Fig. 5F). These values were similar to those seen in untreated control pregnant rats. These findings indicate that the beneficial effect of flutamide in DHT + INS-exposed pregnant rats is mediated by suppression of AR signaling in association with changes in implantation-related gene expression in the uterus during pregnancy.

### Flutamide partially protected against the development of uterine stromal cell mitochondrial impairment in DHT + INS-exposed pregnant rats

**Fig. 6:**
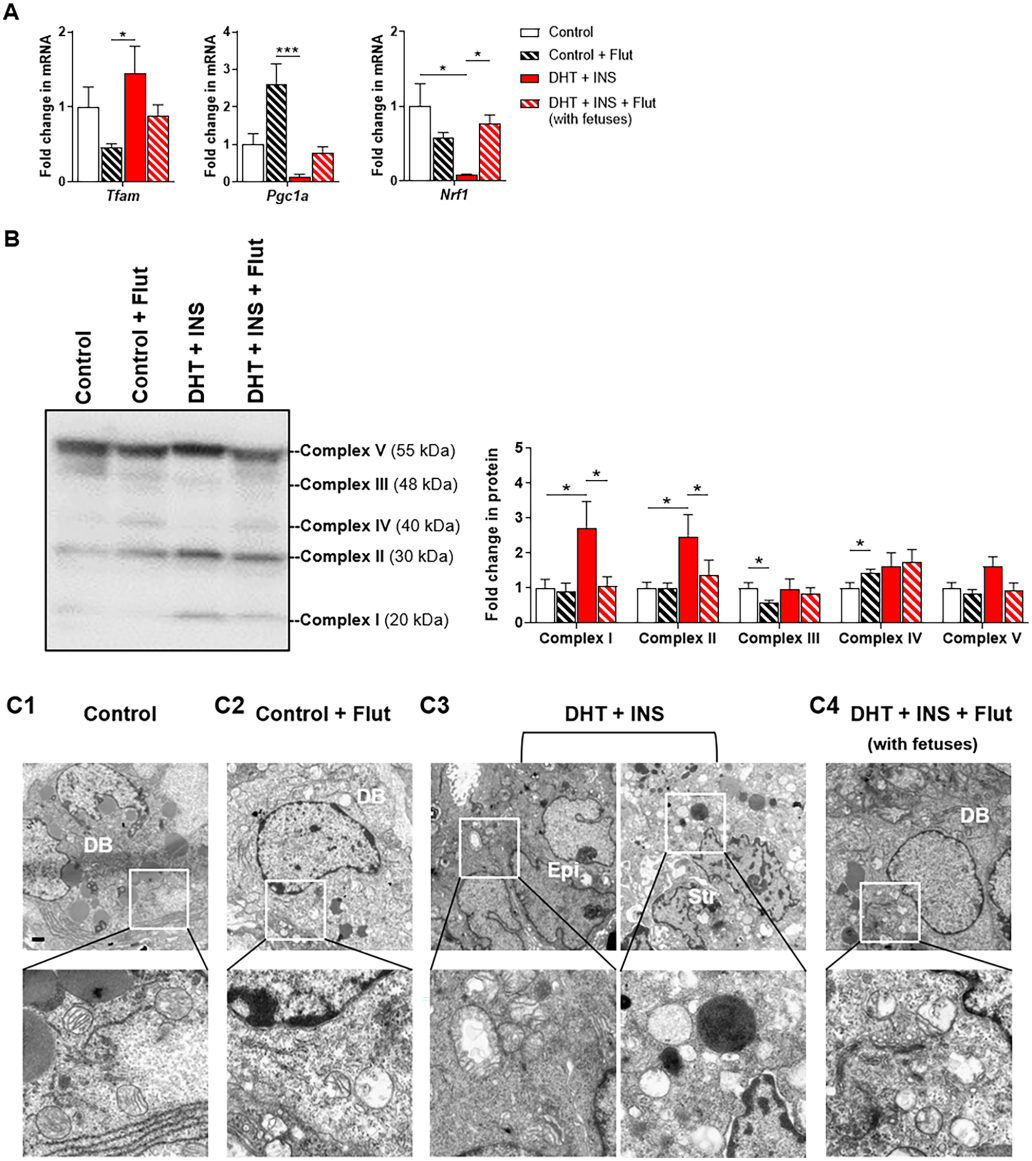
Effects of flutamide on mitochondrial transcriptional activation, uterine oxidative phosphorylation (OXPHOS) protein expression, and mitochondrial morphology in control and DHT + INS-exposed pregnant rats at GD 14.5. After removing the embryos/fetuses and placentas, uterine tissues from control and DHT + INS-exposed pregnant rats treated with flutamide were used for analyzing mRNA levels of *Tfam*, *Pgc1a*, and *Nrf1* by qPCR (**A**, n = 5–7/group), and the relative protein abundance of O_2_-dependent mitochondrial OXPHOS subunits was determined by Western blotting (B, Complexes I–V, 5–6/group). The size representation in kDa, as determined by a molecular weight ladder, is shown to the right. In all plots, data are presented as means ± SEM (vs. Control vehicle values). Statistical tests are described in the “Materials and methods” section, and differences between the groups are reported as * *P* < 0.05, ** *P* < 0.01, and *** *P* < 0.001. Uterine ultrastructural analysis by TEM in control (C1–C2) and DHT + INS-treated (C3–C4) pregnant rats treated with flutamide (C2 and C4). Images are representative of two tissues per group, and enhanced magnifications are shown in the lower panel of each photomicrograph. DB, decidual basalis; Epi, epithelial cells; Str, stromal cells. Scale bars (2 μm) are indicated in the photomicrographs

Due to the changes in gravid uterine mitochondrial function and homeostasis in response to hyperandrogenism and insulin resistance [29], we asked whether flutamide might rescue the mitochondrial morphological and functional defects in the gravid uterus induced by DHT + INS exposure. As shown in Fig. 6A, while flutamide treatment did not significantly impact *Tfam*, *Pgc1a*, or *Nrf1* mRNA expression in control pregnant rats, it significantly increased uterine *Nrf1* mRNA expression in DHT + INS-exposed pregnant rats with fetuses. Although flutamide treatment did not affect *Tfam* or *Pgc1a* mRNA in DHT + INS-exposed pregnant rats, those values were now similar to untreated control pregnant rats (Fig. 6A). The mitochondrial oxidative phosphorylation system is composed of five multi-subunit enzymatic complexes [54], and using Western blotting, we found that flutamide treatment decreased Complex III and increased Complex IV protein levels in control pregnant rats (Fig. 6B). Complex I and II abundances were increased in DHT + INS-exposed pregnant rats, and these effects were abolished by flutamide treatment (in the DHT + INS with fetuses group) (Fig. 6B). Furthermore, TEM analysis showed that compared to controls with normal mitochondrial ultrastructure (Fig. 6C1), DHT + INS-exposed pregnant rats exhibited swollen stromal mitochondria with collapsed and poorly defined tubular cristae (Fig. 6C3). Despite the observation of shrunken/fewer swollen mitochondria in the decidual stromal cells, less electron-dense mitochondria with missing and disorganized cristae were still detected in the DHT + INS-exposed pregnant rats treated with flutamide (Fig. 6C4). These mitochondrial ultrastructural changes were also evident in control rats treated with flutamide (Fig. 6C2).

### Pregnant rats exposed to DHT and INS or DHT alone exhibit ovarian alterations in contrast to flutamide treatment

The impact of DHT and INS on ovarian weight in pregnant rats exposed to DHT and/or INS from GD 4.5 to GD 14.5 was determined (Suppl Fig. 6). We did not find any significant differences for any of the experimental groups from GD 4.5 to GD 10.5; however, we found that on GD 14.5, the ovarian weight was significantly decreased in DHT + INS- and DHT-exposed pregnant rats compared to control and INS-exposed pregnant rats. The corpus lutea (CL) produce several hormones for successful implantation and pregnancy maintenance. Histological analyses and CL counting further revealed that in the ovaries of DHT + INS-exposed pregnant rats, the proportion of the CL number (*n* < 5) per ovary was increased to 55.6% compared to control rats (11.1%). After flutamide treatment, the decreased proportion of the CL number (*n* < 5) per ovary was evident in DHT + INS-exposed pregnant rats (28.6%), similar to that observed in control rats (30.0%) (Suppl Fig. 7A, B). There results suggest that impaired ovarian function might lead to uterine AR-mediated abnormal implantation in pregnant rats with hyperandrogenism.

## Discussion

There are elevated circulating androgen levels in PCOS patients also during pregnancy [4, 5, 25, 26], and PCOS patients have an increased incidence of adverse reproductive outcomes, including pregnancy loss, compared to healthy women [4–6]. Androgen responsiveness is controlled primarily by AR expression [21], which has been implicated in endometrial dysfunction in non-pregnant PCOS patients [24, 28]. However, major knowledge gaps remain regarding whether AR is regulated in the gravid uterus during normal pregnancy establishment, and if so to what extent the effects of aberrant uterine AR lead to PCOS-induced adverse pregnancy outcomes. In this study, we show that AR is differentially expressed in all cell types of the gravid uterus during early and mid-gestation, and we show that the stage-dependent decrease in AR protein abundance is correlated with *Spp1*, *Prl*, *Igfbp1*, and *Hbegf* mRNA expression. This suggests that AR signaling has a pivotal role in embryo implantation. Our previous study in which we exposed rats to DHT and/or INS between GD 7.5 and GD 14.5 (mid-gestation) showed that the expression of genes that are involved in endometrial receptivity and decidualization were aberrant in the gravid uterus [29]. The present study shows that the AR protein abundance is consistently higher in pregnant rats exposed to DHT and INS together or to DHT alone compared to control pregnant rats and to pregnant rats exposed to INS from GD 0.5 to GD 14.5. Moreover, we show that elevated AR protein abundance is accompanied by a lack of implantation likely due to the aberrant expression of genes involved in endometrial receptivity and decidualization in pregnant rats exposed to DHT and INS or to DHT alone, whereas exposure to INS alone affected these parameters to a lower degree. In both human and rodent studies [6], in vivo exposure and in vitro stimulation of androgens not only are associated with, but also directly regulate, the endometrial receptivity and decidualization-related gene expression, and they influence cell oxidative stress, proliferation, and apoptosis. Previous studies demonstrated that female AR knockout mice exhibited smaller uteri with an abnormal estrous cycle, impaired placental development, and reduced fertility over time [55, 56]. Based upon the aforementioned evidence, it was speculated that the causality of androgen-induced uterine dysfunction and subfertility might be the result of two opposite extremes, namely AR overexpression and AR knockdown. However, the idea of AR-related subfertility has been challenged by the observations that a mouse model with an in-frame deletion of exon 3 of the *Ar* gene [57] presents with decreased ovarian weight and ovulation rate, increased unhealthy antral follicle number and ovarian T level, and reduced litter size [57, 58] as well as abnormal neuroendocrine function [58]. According to this mouse model, the lack of non-functional AR (which involves the ligand-induced nuclear localization of a transcriptionally inactive AR protein) does not affect uterine-related reproductive capabilities (e.g., damaged implanted embryo viability, and perturbed pup survival and gestational length) [58]. These results suggest that uterine AR signaling might not be essential for normal pregnancy and fertility. However, similar to our pregnant PCOS-like rats, these AR knockout mice exhibit a significant decrease in ovarian weight and in CL and implantation site numbers, as well as reduced uterine weight and reduced endometrial area [58]. Because our present study documents the complexity of the disturbances to the androgen-AR signaling axis that result in impaired endometrial receptivity and decidualization processes, future investigation should determine whether elevated androgen levels, increased AR distribution and activity, and/or impaired androgen-AR signaling mechanisms have an impact on uterine-related implantation failure and infertility using uterine tissue/cell-specific AR knockout mice.

A significant strength of this study is the mechanistic findings from chronic administration of flutamide for treating infertility in pregnant rats under conditions of hyperandrogenism and insulin resistance (resulting from co-exposure to DHT and INS). The biological effects of flutamide are mediated through competitively inhibiting the binding of androgens to the AR [59], and at the molecular and functional levels, our data demonstrate that treatment with flutamide from pre-implantation, through implantation, to post-implantation suppresses the increased AR and p21^WAF1/CIP1^ protein abundances in the pregnant uterus co-exposed to DHT and INS. Further, we show that treatment with flutamide effectively reverses DHT + INS-induced deficiencies in endometrial receptivity and decidualization and subsequently increases the numbers of viable fetuses and restores fertility. Likewise, in pregnant mice flutamide treatment elicited a marked reversal of testosterone-induced decreases in decidualization-related gene expression, and it decreased the number of resorbed embryos during implantation [60]. Furthermore, in women with PCOS, long-term anti-androgen therapy is associated with decreased testosterone levels and improved ovulatory function [61]. There is evidence from a Swedish nationwide register-based cohort study indicating that early treatment with different anti-androgens, including flutamide, is correlated with a higher chance of childbirth in PCOS patients after spontaneous conception [20]. Therefore, we reasoned that flutamide regulates PCOS-related poor pregnancy outcomes possibly by inhibiting AR-mediated reductions in endometrial receptivity and decidualization, which are required for implantation during early pregnancy. However, the existing literature is contradictory regarding the beneficial effects of anti-androgens on decidualization and implantation. For instance, during the implantation window treatment with hydroxyflutamide, an active metabolite of flutamide, suppressed decidualization and delayed implantation in pregnant and pseudopregnant rats [62]. We speculate that the gravid uterus might have a different time frame in response to or in the regulation of endometrial receptivity and decidualization processes that can change its sensitivity to treatment with different anti-androgens. In line with this speculation, we show that in control pregnant rats flutamide treatment resulted in significantly decreased *Lif* and *Il11* mRNA expression, although no negative effect on fetal number or fertility was observed. Furthermore, as we demonstrated in this study, the effectiveness of flutamide on fertility is most clearly seen in pregnant rats co-exposed to DHT and INS. Because of increased P450 aromatase mRNA expression in the PCOS endometrium [63], our findings do not rule out the possibility that flutamide might affect decidualization and implantation through other mechanisms (e.g., through interactions with estrogen receptor) in vivo [64], and this requires future study.

**Fig. 7:**
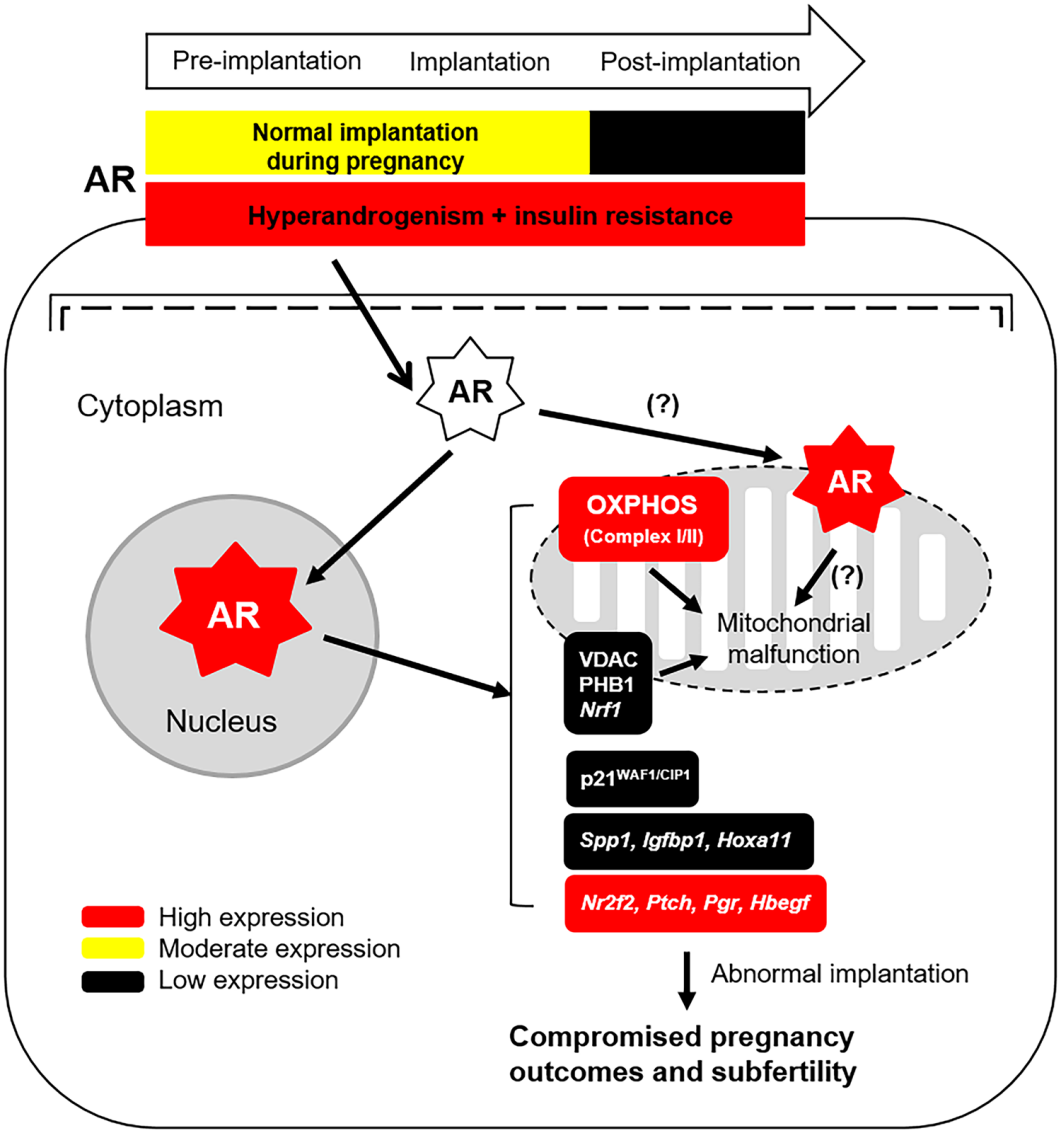
A schematic representation illustrating our working hypothesis regarding the regulation and activation of gravid uterine AR signaling pathways after combined exposure of hyperandrogenism and INS resistance. The signaling pathway depicted is based on the findings of the current study. There is evidence that cytosolic AR is translocated to and/or exists in the mitochondrion in several cell types and thus might exert effects downstream of the hyperandrogenism, thus leading to mitochondrial dysfunction. Further studies are required to determine whether PCOS-induced uterine cell defects are due to the mitochondrial AR actions during pregnancy

Emerging evidence suggests that mitochondrial dysfunction is one of the etiological factors in the pathogenesis of PCOS [32]. Recently, we demonstrated that during mid-gestation the combined exposure to DHT and INS mimics the in vivo hyperandrogenism and insulin resistance seen in humans and increases mitochondrial abnormalities in the gravid uterus and placenta [29–31]. In this study, our findings extend our understanding of the mechanisms governing AR-regulated mitochondrial function in the gravid uterus. As noted, the presence of flutamide results in increased *Nrf1* mRNA expression and decreased levels of several mitochondrial-related proteins (i.e., VDAC, PHB1, and mitochondrial respiratory Complexes I and II) along with decreased AR and p21^WAF1/CIP1^ levels in rats co-exposed to DHT and INS. However, flutamide did not restore the mitochondrial structural defects that result from exposure to DHT and INS. AR is predominantly characterized as a nuclear receptor that regulates the transcription of target genes [21]. In addition to the translocation of AR from the cytoplasm to the nucleus in cells [21], the mitochondrial distribution of endogenous AR has been demonstrated in human sperm, prostate adenocarcinoma cells, and skeletal muscle cells [65–67]. Although we cannot rule out the possibility that in vivo mitochondrial AR alone contributes to PCOS-induced uterine cell dysfunction, in vitro studies have shown that flutamide is toxic to mitochondria by reducing respiratory Complex I activity [68, 69]. Therefore, it is likely that the over-activation of nuclear AR signaling is responsible for some, if not all, of the gravid uterine dysfunction that results in infertility in pregnant rats under conditions of hyperandrogenism and insulin resistance. The regulation of AR activation in the nuclear and extra-nuclear compartments in uterine cells under physiological conditions and disease states is likely to be more complex than we previously thought, and additional studies are needed to determine the mitochondrial AR localization and functional relevance in uterine cells (Fig. 7).

For a better understanding of the link between elevated endometrial inflammation and PCOS, we recently reported the increases in infiltrated endometrial immune cells in non-pregnant PCOS patients with hyperandrogenism and insulin resistance [28, 70]. Similarly, our morphological analysis indicates that co-exposure to DHT and INS increases the numbers and infiltration of immune cells into the uterine endometrium in pregnant rats. Furthermore, this uterine pathological phenomenon is also seen in DHT and INS co-exposed pregnant rats that receive flutamide treatment but fail to complete a normal pregnancy. Our data suggest that the inhibition of DHT + INS-induced uterine inflammation is at least partly dependent on decreased AR protein and blocked AR signaling. It has long been recognized that decidual immune cells such as uterine natural killer (uNK) cells and macrophages contribute to appropriate embryo implantation and successful pregnancy, and alterations in these cell populations may be associated with pregnancy-related complications [71]. Substantial evidence indicates that PCOS patients have aberrant/altered circulating levels of immune cells and uNK cell abundance in the uterus during the secretory phase of the menstrual cycle [8, 72]. In pregnant mice during post-implantation, treatment with testosterone propionate decreased the numbers of uNK cells in the uterus, and the additional treatment with flutamide restored the normal distribution of uNK cells [60]. Although the different types of immune cells, including NK cells and macrophages, in humans and rodents express AR [73], whether decidual immune cells also express AR during pregnancy remains unclear [22]. Because the endometrial epithelial cells and decidual and non-decidual stromal cells are the major cell types that express AR [24, 51, 52, 74], we hypothesized that the uterine epithelial and stromal/decidual cells modulate immune cell-mediated inflammatory responses through paracrine-regulated AR signaling pathways that are activated under conditions of hyperandrogenism and insulin resistance.

Clinical and pre-clinical studies support the notion that high levels of maternal androgens (T, A4, and/or DHT) are associated with PCOS [25, 26, 29, 30, 42, 43] and early pregnant loss [30, 75]. Although direct androgenic actions through the AR can only be mediated by T and DHT in tissues/cells [21], in vivo and in vitro studies show that androgens that operate in a ligand-dependent and cell-specific manner modulate uterine cell survival. For instance, in nonpregnant rodents in vivo exposure to DHT induces epithelial cell proliferation and promotes uterine tissue growth [76, 77], which is mediated by the activation of stromal estrogen receptor α signaling [77, 78]. Despite not always being the case [79], in vitro treatment with A4 can increase cell proliferation and decrease apoptosis in human endometrial stromal cells [80, 81]. In contrast, treatment with T and DHT inhibits cell proliferation in human endometrial stromal cells regardless of whether the decidualization occurs or not [81–83]. It is currently unknown whether endogenous androgens regulate uterine cell proliferation in pregnant rats; however, chronic exposure of DHT alone or in combination with INS from GD 7.5 to GD 14.5 increases ferroptosis and decreases apoptosis in the gravid uterus [31]. Of note, the trimester-dependent alteration of androgen levels has been observed in humans during normal pregnancy [19, 26]. Given that exposure to high doses of DHT results in elevated maternal T and A4 levels, which are similar to pregnant PCOS patients with hyperandrogenic conditions [25, 26, 42, 43], it is likely that a crucial regulatory balance between the physiological roles and the detrimental effects of maternal androgens may determine cell proliferation and cell death in the uterus during pregnancy.

In summary, the present findings support the hypothesis that the in vivo gestational stage-dependent expression and regulation of uterine AR protein abundance contributes to embryo implantation success. The effect of the anti-androgen flutamide in the gravid uterus suggests that aberrant AR expression and its signaling dysregulates the expression of genes and proteins that are involved in endometrial receptivity and decidualization as well as in mitochondrial function and that together these result in compromised fertility (Fig. 7). While PCOS is one of the most frequent causes of infertility in women of reproductive age, it has been proposed that the causes for implantation failure in PCOS patients might be due to endometrial defects as indicated by the dysregulation of the expression of proteins required for implantation in the human endometrium [7, 8]. Future studies should be directed toward determining whether pregnant PCOS patients exhibit similar abnormal expression patterns of AR and endometrial receptivity and decidualization-related molecules in the gravid endometrium that cause implantation failure. Overall, our current results provide new insights into the reproductive function of AR in the gravid uterus and provide a mechanistic rationale for treating PCOS patients with anti-androgens for preventing pregnancy loss and for improving fertility under conditions of hyperandrogenism and insulin resistance.

## Supplementary Information

Below is the link to the electronic supplementary material.

Supplementary file1 (PDF 1812 KB)

## Data Availability

Data and material are available upon request to the corresponding author.
