# Supplementary material for: Increased uterine androgen receptor protein abundance results in implantation and mitochondrial defects in pregnant rats with hyperandrogenism and insulin resistance
Source: J Mol Med (Berl). 2021 Jun 28;99(10):1427–46. doi: 10.1007/s00109-021-02104-z (PMC8455403; doi:10.1007/s00109-021-02104-z)
Supplement: Supplementary file 1 — Supplementary file1 (PDF 1812 KB) [file 109_2021_2104_MOESM1_ESM.pdf]

Supporting Data

Deciphering the role of uterine AR in PCOS during early pregnancy

Yuehui Zhang[1,2], Min Hu[2,3,4], Fan Yang[1], Yizhuo Zhang[1], Shuting Ma[1], Dongqi Zhang[1], Xu Wang[1], Amanda Nancy Sferruzzi-Perri[5], Xiaoke Wu[1], Mats Brännström[6], Linus R Shao[2], and Håkan Billig[2]

[1] Department of Obstetrics and Gynecology, Key Laboratory and Unit of Infertility in Chinese Medicine, First Affiliated Hospital, Heilongjiang University of Chinese Medicine, 150040 Harbin, China

[2] Department of Physiology/Endocrinology, Institute of Neuroscience and Physiology, The Sahlgrenska Academy, University of Gothenburg, 40530 Gothenburg, Sweden

[3] Department of Traditional Chinese Medicine, The First Affiliated Hospital of Guangzhou Medical University, 510120 Guangzhou, China

[4] Institute of Integrated Traditional Chinese Medicine and Western Medicine, Guangzhou Medical University, 510120 Guangzhou, China

[5] Centre for Trophoblast Research, Department of Physiology, Development and Neuroscience, University of Cambridge, Cambridge, CB2 3EG, UK

[6] Department of Obstetrics and Gynecology, Sahlgrenska University Hospital, Sahlgrenska Academy, University of Gothenburg, 41345 Gothenburg, Sweden

Key findings: Hyperandrogenism and insulin resistance perturb the implantation process and mitochondrial dysfunction through the defective androgen receptor signaling in the rat gravid uterus

Supporting data include two Supplemental Tables, and 7 Supplemental Figures

Supporting Data

Supplemental Table 1. Sequences of primer pairs used for qRT-PCR measurement.

Gene		Primer	Product Size
Lif	Forward	CGCCCAACATGACGGATTTC	226 bp
	Reverse	TTGTTGCACAGACGGCAAAG	
Nr2f2	Forward	GTCGCCTTTATGGACCACAT	145 bp
	Reverse	CGTGGGCTACATCAGACAGA	
Pc6	Forward	GTGTGAGAATGGGTCGGAGA	149 bp
	Reverse	TTTCTTTCCACTTTCGGCCG	
Ptch	Forward	GAACAAGCAACTTCCCCAAA	190 bp
	Reverse	AATGTCGATGGGCTTGTCTC	
Spp1	Forward	CTGAAGCCTGACCCATCTCA	143 bp
	Reverse	TCGTCGTCATCATCGTCCAT	
Prl	Forward	CAAGAAGAAGGGGCCAACCT	100 bp
	Reverse	CTGGTGGTGACTGTCCCTTC	
Igfbp1	Forward	TGTACTAGAACCTGCCGCAC	126 bp
	Reverse	AGCAGCTGTTCCTCTGTCAT	
Pgr	Forward	GGTCTAAGTCTCTGCCAGGTTTCC	182 bp
	Reverse	CAACTCCTTCATCCTCTGCTCATTC	
Hoxa10	Forward	TCCGAAAACAGTAAAGCCTCTC	127 bp
	Reverse	GCGTCTGGTGCTTCGTGTAA	
Hoxa11	Forward	GACTCCCTACCTACCTTGGC	100 bp
	Reverse	GCAACCACTGTACATGTCGG	
Il11	Forward	GACTCCCTACCTACCTTGGC	100 bp
	Reverse	GCAACCACTGTACATGTCGG	
Hbegf	Forward	GCTCTTCCACCTGGCTCAAT	120 bp
	Reverse	CACAACCCACCCTGGGATAC	
Tfam	Forward	ACAAAGAAGCTGTGAGCAAGTA	99 bp
	Reverse	GTGCTTTCTTCTTTAGGCGTTTC	
Pgc1a	Forward	GTGGATGAAGACGGATTGCC	219 bp
	Reverse	GGTGTGGTTTGCATGGTTCT	
Nrf1	Forward	GGAAACTCAGAGCCACATTAGA	109 bp
	Reverse	GCGCCAAACACCTTAAAGAC	
Gapdh	Forward	TCTCTGCTCCTCCCTGTTCTA	121 bp
	Reverse	GGTAACCAGGCGTCCGATAC	
Actb	Forward	CGCGAGTACAACCTTCTTGC	70 bp
	Reverse	CGTCATCCATGGCGAACTGG	

Lif, leukemia inhibitory factor; *Nr2f2*, nuclear receptor subfamily 2 group F member 2; *Pc6*, protein convertase 5/6; *Ptch*, patched; *Spp1*, osteopontin/secreted phosphoprotein 1; *Prl*, prolactin; *Igfbp1*, insulin-like growth factor binding protein 1; *Pgr*, progesterone receptor; *Hoxa10*, homeobox A10; *Il11*, interleukin-11; *Hbegf*, heparin-binding EGF-like growth factor; *Tfam*, mitochondrial transcription factor a; *Pgc1a*, peroxisome proliferative activated receptor gamma coactivator 1 alpha; *Nrf1*, nuclear respiratory factor 1; *Gapdh*, glyceraldehyde-3-phosphate dehydrogenase; *Actb*, beta-actin.

Supporting Data

Supplemental Table 2. Antibodies: species, clone/catalog number, method, dilution, and source.

Antibody	Species	Clone / Cat. No.	kDa	Method	Dilution	Source
AR	Rabbit	EPR1535(2) /133273	98	WB<br>IHC	1:1000<br>1:200	Abcam (Cambridge, UK)
pan-Cytokeratin	Mouse	C-11/P2871	45-59	WB	1:1000	Sigma-Aldrich (St. Louis, MO)
Vimentin	Rabbit	D21H3/5741	57	WB	1:1000	Cell Signaling Technology (Danver, MA)
α-SMA	Mouse	1A4/A5228	42	WB	1:1000	Sigma-Aldrich
$p21^{WAF1/CIP1}$	Mouse	F-5/6246	21	WB	1:200	Santa Cruz Biotechnology (Heidelberg, Germany)
VDAC	Rabbit	D73D12/4661	32	WB	1:1000	Cell Signaling Technology
PHB1	Rabbit	2426	32	WB	1:1000	Cell Signaling Technology
Total OXPHOS	Mouse	110413	I 20<br>II 30<br>III 48<br>IV 40<br>V 55	WB	1:500	Abcam

AR, androgen receptor; α-SMA, α-smooth muscle actin; VDAC, voltage-dependent anion channel; PHB1, prohibitin 1; Total OXPHOS, total oxidative phosphorylation; WB, western blot; IHC, immunohistochemistry.

Supporting Data

Suppl Figure legend

Suppl Figure 1. Characterization of the specificity of the AR antibody. The signal intensity of cellular AR decreases with increasing antibody concentration. Using the uterus from rats on GD 4.5 for immunohistochemical staining, an antibody against AR was used to determine the proper concentration of antibody (1:200 dilution). Representative images of AR antibody staining at different dilutions are shown (1—5). The same concentration of rabbit IgG instead of the primary and secondary antibodies was used as the negative control (6). Lm, longitudinal myometrium; Cm, circular myometrium. Scale bars (100 μm) are indicated in the photomicrographs.

Suppl Figure 2. Localization of AR protein in uteri collected from control pregnant rats at GD 7.5. Hematoxylin and eosin (H&E) counterstaining and negative control serial sections are shown in the left and right panels. Due to continuing proliferation and differentiation of the stromal fibroblast cells into decidual cells, the decidual cells are larger in the PDZ and SDZ than those in DB area. Images for AR immunostaining (the middle panel) are representative of 8 tissue replicates. GD, gestational day; DB, decidual basalis; E, embryo; PDZ, primary decidual zone; SDZ, secondary decidual zone; Cm, circular myometrium; Lm, longitudinal myometrium; Le, luminal epithelial cells; Ge, glandular epithelial cells; Str, stromal cells. Scale bars (100 μm) are indicated in the photomicrographs.

Suppl Figure 3. Localization of AR protein in uteri collected from control pregnant rats at GD 10.5. H&E counterstaining is shown in the left panel. Images for AR immunostaining (the right panel) are representative of 8 tissue replicates. Ge, glandular epithelial cells; Lm, longitudinal myometrium; DB, decidual basalis; SDZ, secondary decidual zone. Scale bars (100 μm) are indicated in the photomicrographs.

Suppl Figure 4. Localization of AR protein in uteri collected from control pregnant rats at GD 14.5. H&E counterstaining and negative control serial sections are shown in the left and right panels, respectively. Images for AR immunostaining (the right panel) are representative of 8 tissue replicates. Ut, uterus; P, placental disc; F, fetus; MT, mesometrial triangle; MD, mesometrial decidua; BZ, basal zone; LZ, labyrinth zone; GC, glycogen cells; Sp, spongiotrophoblast cells; Cp, cytotrophoblast cells; Sy, syncytiotrophoblast cells; MV, maternal vessel; FV, fetal vessel. Scale bars (100 μm) are indicated in the photomicrographs.

Suppl Figure 5. Treatment with flutamide only partially restores fertility in DHT+INS-exposed pregnant rats. Histological analysis by H&E counterstaining showing that no fetuses were found in DHT+INS-exposed pregnant rats treated with flutamide. The yellow arrowheads indicate infiltrated immune cells in the endometrial gland. Images are representative of 3 tissue replicates. M, myometrium; En, endometrium; G, gland. Scale bars (100 μm) are indicated in the photomicrographs.

Suppl Figure 6. Effects of DHT and INS on ovarian weight in pregnant rats from GD 4.5 to GD 14.5. Comparison of ovarian weight in pregnant rats treated with and without DHT and/or INS (n = 8/group). Data are presented as means ± SEM. Statistical tests are described in the Materials and Methods, and differences between the groups are reported as * $P < 0.05$.

Suppl Figure 7. Effects of flutamide on ovarian morphology and corpus lutea number in control and DHT+INS-exposed pregnant rats at GD 14.5. Histological analysis by H&E staining (A) and quantification of the total numbers of corpus lutea (B) in vehicle control and DHT+INS-exposed pregnant rats treated with flutamide (n = 7—10/group). Scale bars (100 μm) are indicated in the photomicrographs.

Suppl Fig. 1

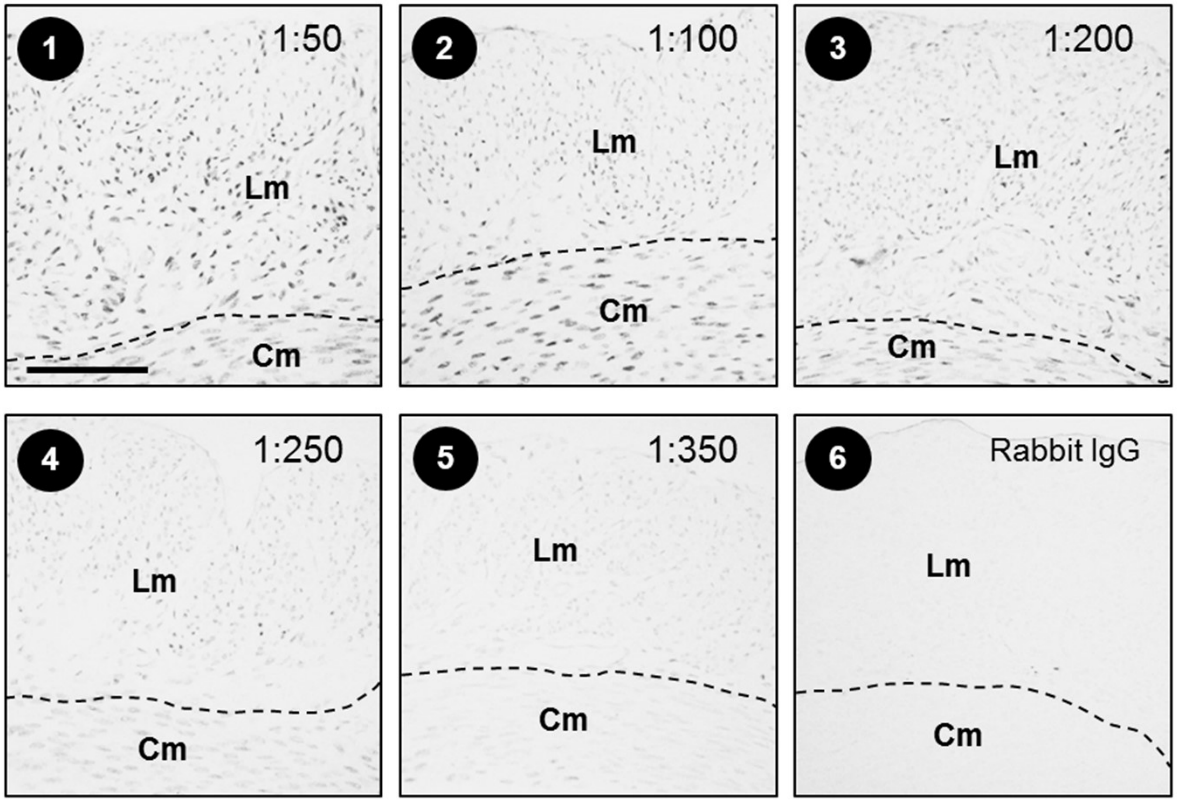

1
1:50
Lm
Cm
2
1:100
Lm
Cm
3
1:200
Lm
Cm
4
1:250
Lm
Cm
5
1:350
Lm
Cm
6
Rabbit IgG
Lm
Cm

Suppl Fig. 2

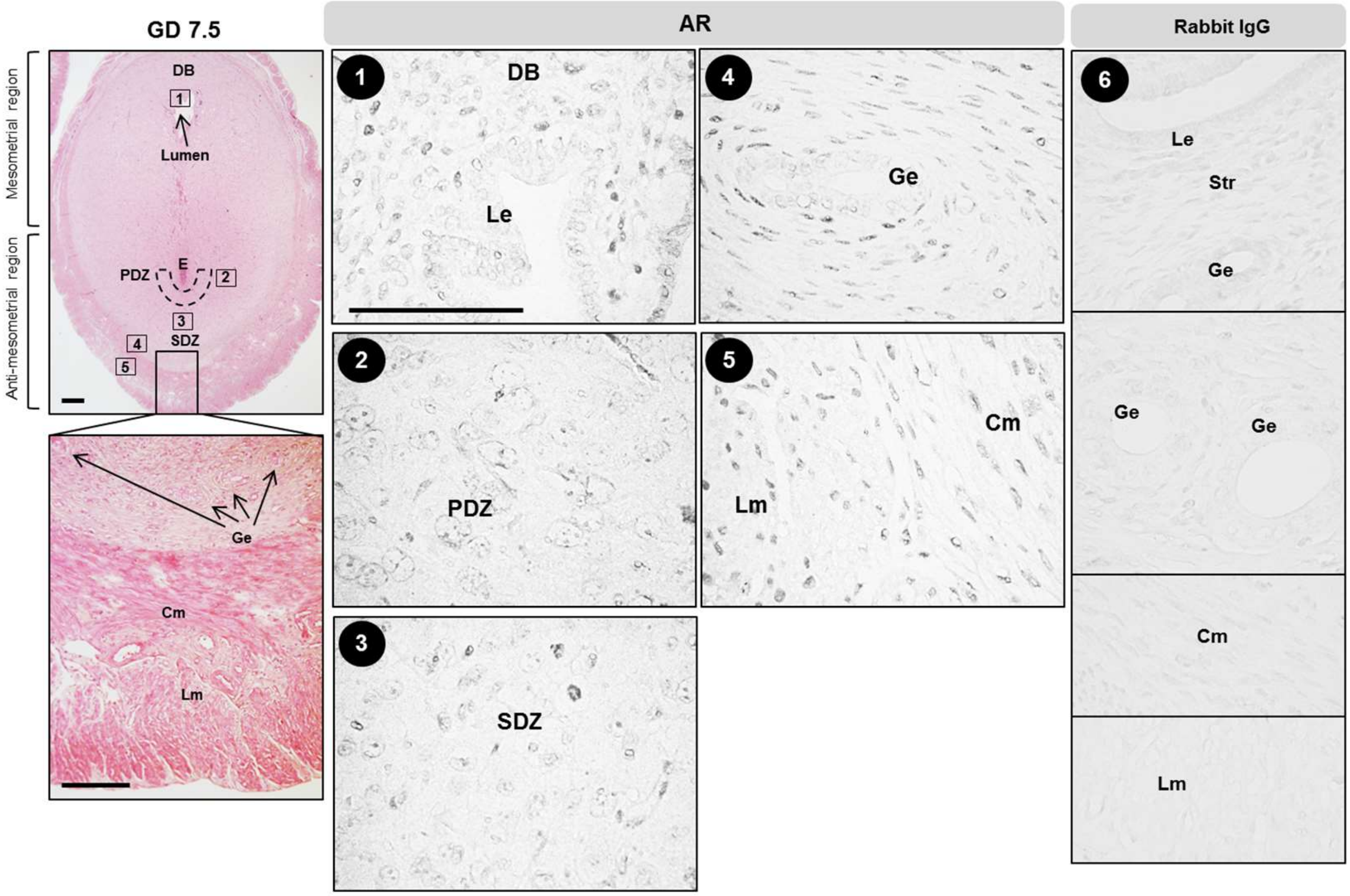
GD 7.5
AR
Rabbit IgG
Mesometrial region
Anti-mesometrial region
DB
1
Lumen
E
PDZ
2
3
SDZ
4
5
Ge
Cm
Lm
1
DB
Le
4
Ge
2
PDZ
5
Cm
Lm
3
SDZ
6
Le
Str
Ge
Ge
Ge
Cm
Lm

Suppl Fig. 3

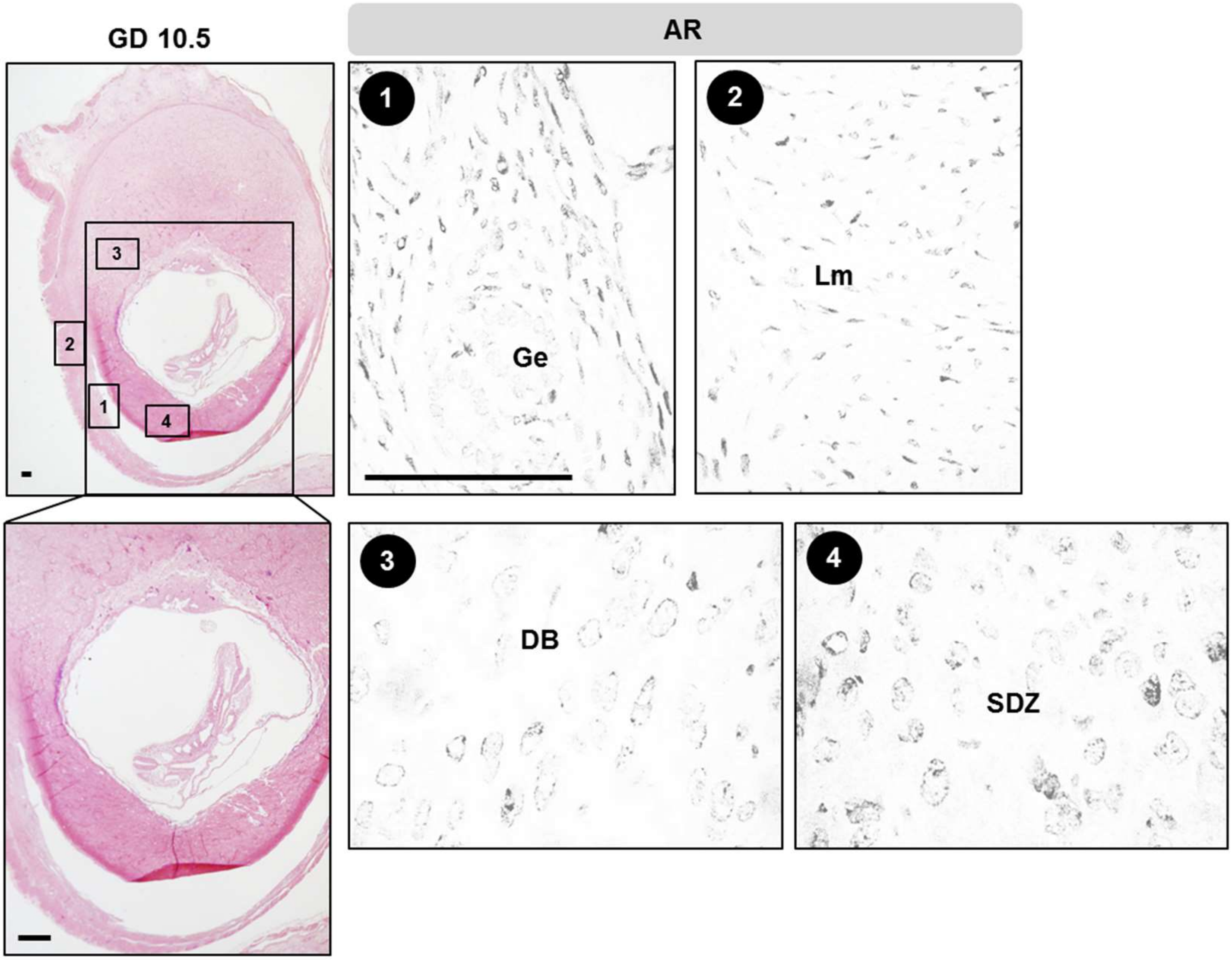

Suppl Fig. 4

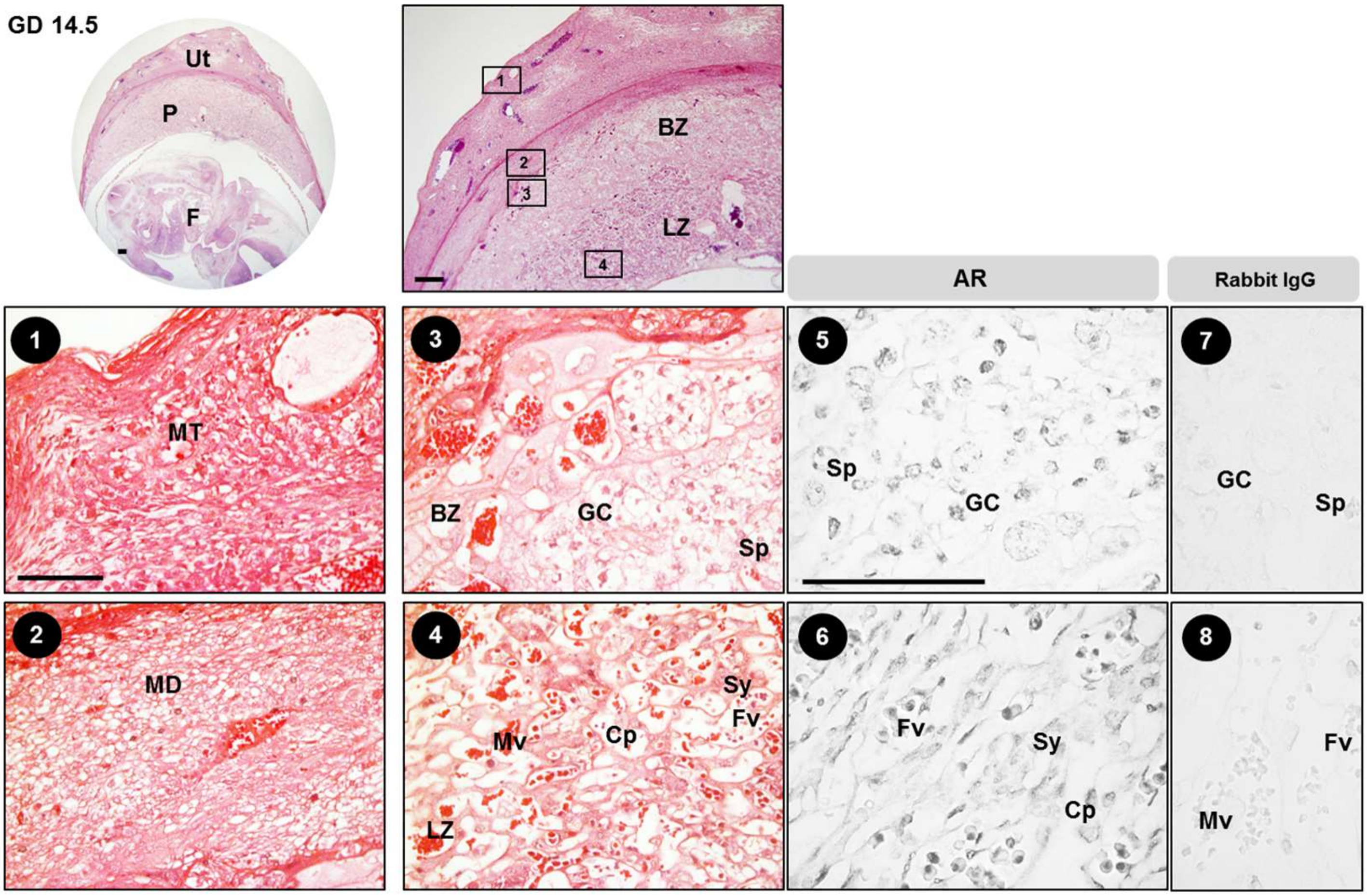

GD 14.5
Ut
P
F
1
2
3
4
BZ
LZ
AR
Rabbit IgG
1
MT
2
MD
3
BZ
GC
Sp
4
Sy
Fv
Mv
Cp
LZ
5
Sp
GC
6
Fv
Sy
Cp
7
GC
Sp
8
Fv
Mv

Suppl Fig. 5

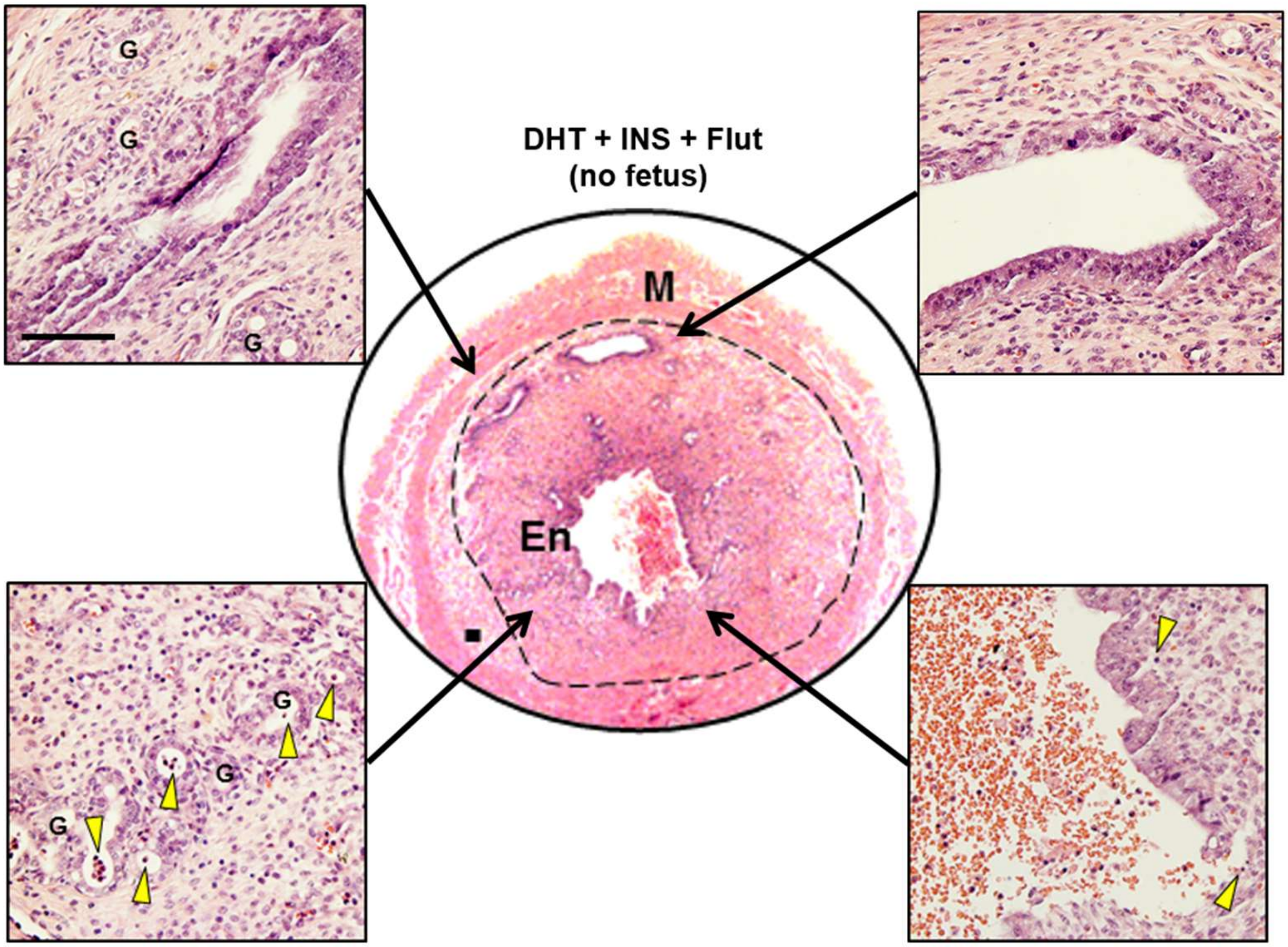

Suppl Fig. 6

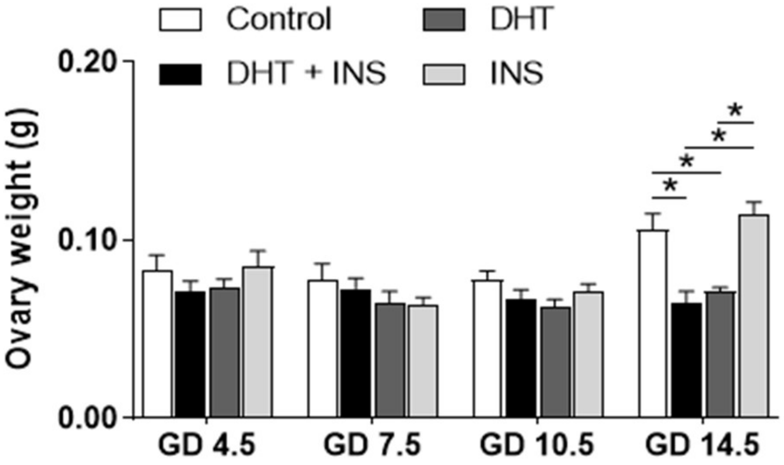

Control
DHT
DHT + INS
INS
0.20
0.10
0.00
Ovary weight (g)
*
*
*
*
GD 4.5
GD 7.5
GD 10.5
GD 14.5

Suppl Fig. 7

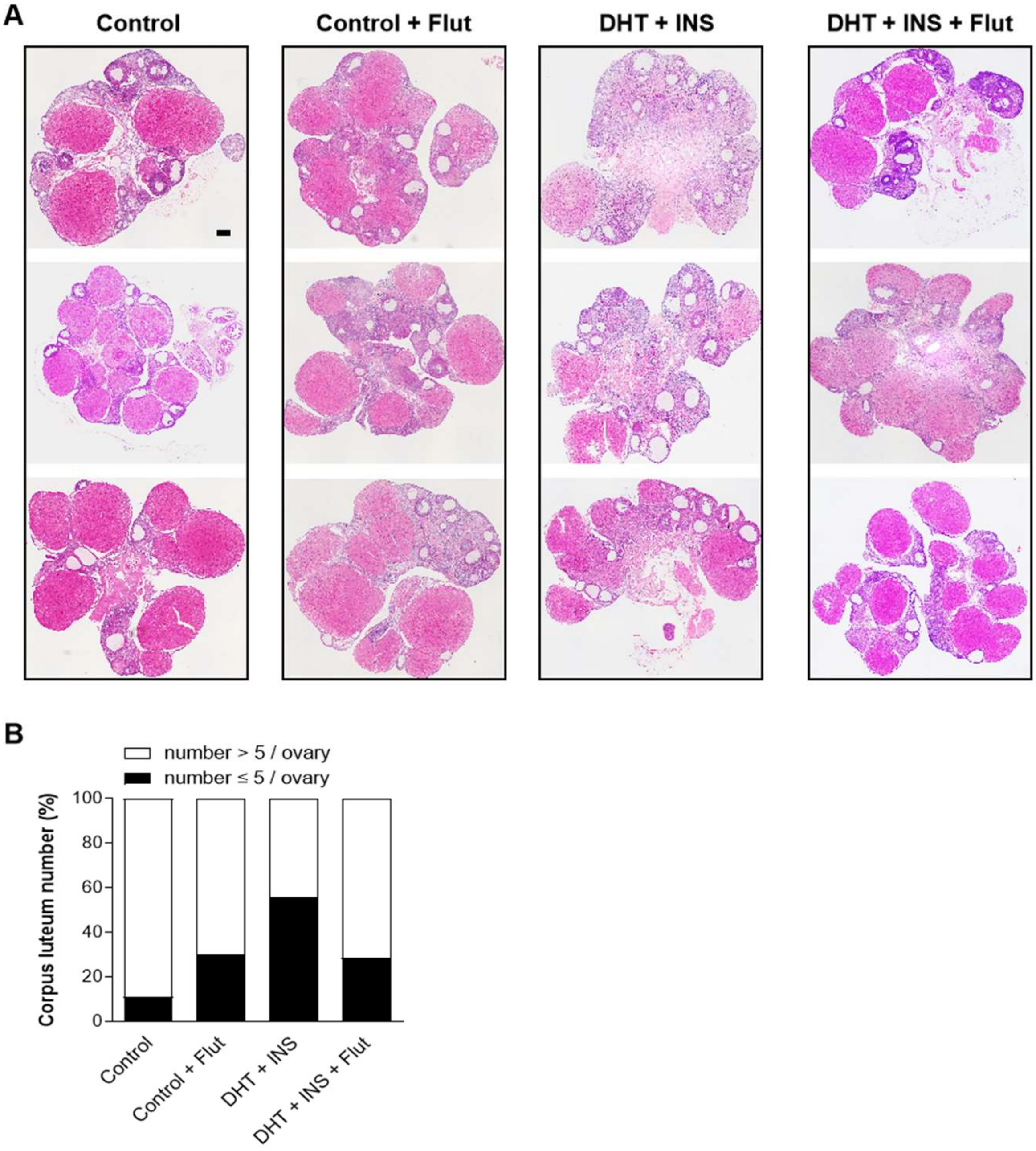
A
Control
Control + Flut
DHT + INS
DHT + INS + Flut
B
number > 5 / ovary
number ≤ 5 / ovary
Corpus luteum number (%)
100
80
60
40
20
0
Control
Control + Flut
DHT + INS
DHT + INS + Flut
